# Injection continuous liquid interface production of 3D objects

**DOI:** 10.1126/sciadv.abq3917

**Published:** 2022-09-28

**Authors:** Gabriel Lipkowitz, Tim Samuelsen, Kaiwen Hsiao, Brian Lee, Maria T. Dulay, Ian Coates, Harrison Lin, William Pan, Geoffrey Toth, Lee Tate, Eric S. G. Shaqfeh, Joseph M. DeSimone

**Affiliations:** ^1^ Department of Mechanical Engineering, Stanford University, Stanford, CA 94305, USA.; ^2^ Department of Radiology, Stanford University, Stanford, CA 94305, USA.; ^3^ Department of Mechanical Engineering, Sungkyunkwan University, Seoul, South Korea.; ^4^ Department of Chemical Engineering, Stanford University, Stanford, CA 94305, USA.; ^5^ Department of Mechanical Engineering, Product Design, Stanford University, Stanford, CA, 94305, USA.; ^6^ Digital Light Innovations, Austin, TX 78728, USA.

## Abstract

In additive manufacturing, it is imperative to increase print speeds, use higher-viscosity resins, and print with multiple different resins simultaneously. To this end, we introduce a previously unexplored ultraviolet-based photopolymerization three-dimensional printing process. The method exploits a continuous liquid interface—the dead zone—mechanically fed with resin at elevated pressures through microfluidic channels dynamically created and integral to the growing part. Through this mass transport control, injection continuous liquid interface production, or iCLIP, can accelerate printing speeds to 5- to 10-fold over current methods such as CLIP, can use resins an order of magnitude more viscous than CLIP, and can readily pattern a single heterogeneous object with different resins in all Cartesian coordinates. We characterize the process parameters governing iCLIP and demonstrate use cases for rapidly printing carbon nanotube–filled composites, multimaterial features with length scales spanning several orders of magnitude, and lattices with tunable moduli and energy absorption.

## Teaser

iCLIP enables rapid creation of 3D complex objects with high-viscosity composite resins and multiple materials simultaneously.

## INTRODUCTION

**Fig. 1.**
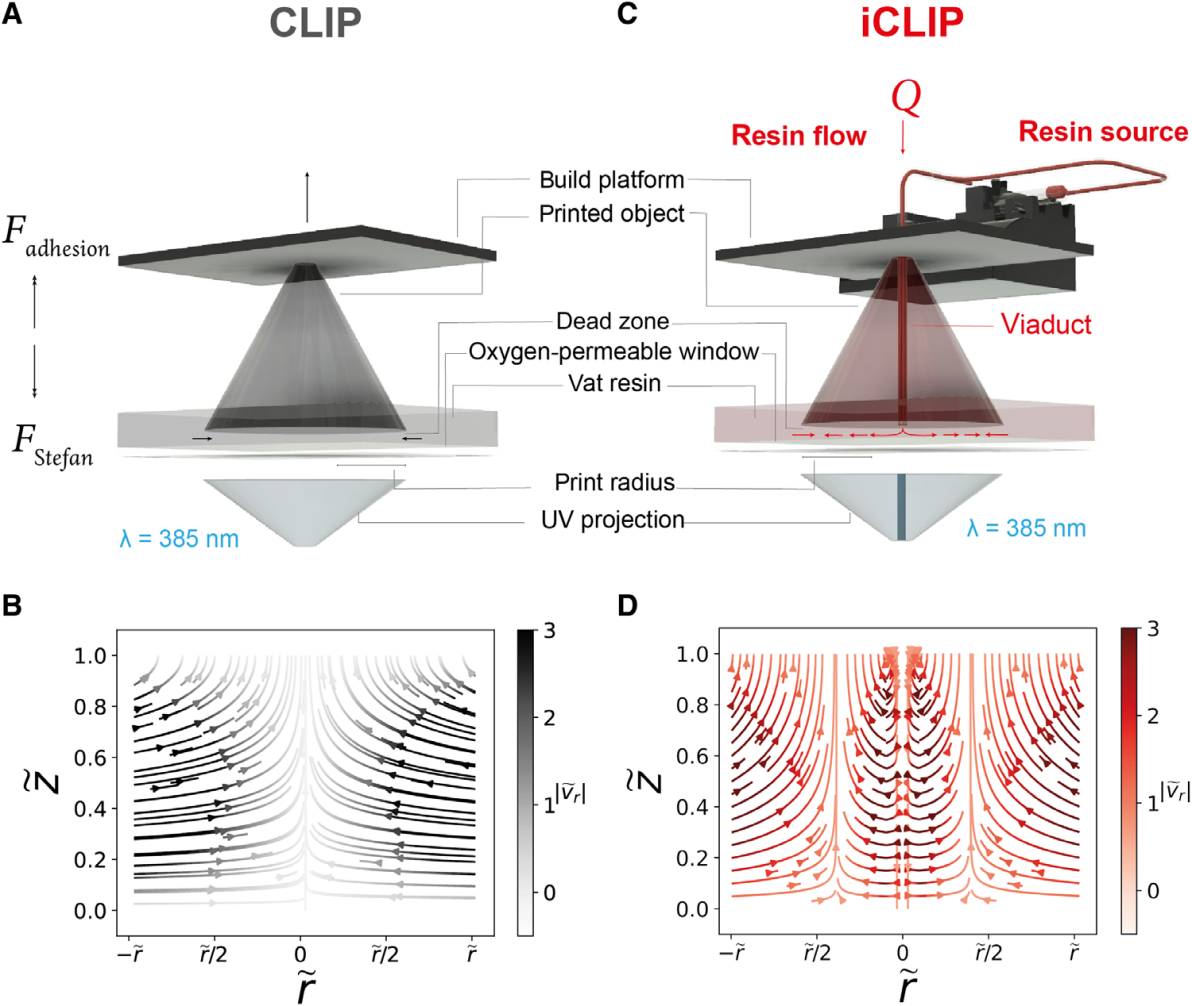
Injection continuous liquid interface production. (**A**) Traditional CLIP process, with force diagram for the printed object and resin flows indicated. (**B**) Analytically derived dead zone velocity fields and pressure gradients from the lubrication theory while printing a cylindrical geometry by CLIP, where $\tilde{z}$ and $\tilde{r}$ are the vertical and radial distances in the dead zone, respectively, and $\tilde{v_{r}}$ is the radial velocity. Darker hues indicate higher-magnitude velocity vectors, and, conversely, lighter hues indicate stagnation zones of low-fluid velocity. (**C**) iCLIP process indicating the flow of the injected resin from a pressurized source through microfluidic ducts engineered within the growing part into the dead zone. (**D**) Analytically derived dead zone velocity fields and pressure gradients from the lubrication theory while printing a cylindrical geometry by CLIP, with continuous injection through a central viaduct.

Ultraviolet (UV)–curable liquid resin–based additive manufacturing (AM) can broadly be divided into three generations of vat photopolymerization (VP) (*1*) and material jetting (MJ). In first-generation VP, also known as stereolithography, a bath containing a single liquid resin is photocured spatioselectively by a scanning laser point source. Second-generation VP, referred to as digital light projection, uses a rapid sequence of projected UV images that span the entire *XY* plane of a bath containing a liquid resin in a single exposure. Often referred to as third-generation VP, continuous liquid interface production (CLIP; Fig. 1A) relies on resin renewal at the build surface through a continuous liquid interface—the dead zone—created by oxygen, a polymerization inhibitor, fed through the oxygen-permeable window beneath the vat (*2*–*4*). CLIP, achievable with multiple patterns of platform movement and UV exposure (*5*) along with different window configurations (*6*, *7*), enables printing at speeds of up to 3000 mm/hour, 25 to 100 times higher than traditional AM methods. While, to date, CLIP has been limited to relatively low-viscosity resins [commercially available resins from Carbon Inc. have viscosities of up to roughly 2500 centipoise (cP)] (*8*), CLIP produces isotropic parts, unlike conventional three-dimensional (3D) printing methods such as fused filament fabrication and powder bed fusion, and has been proven suitable for manufacturing at high volumes and at high resolution for, e.g., biomedical devices (*9*, *10*).

However, CLIP is still notably slower than injection molding. This is due to severe mass transport limitations on resin flow through the thin dead zone (Fig. 1B), inducing Stefan adhesion forces that require delay time to equilibrate the negative dead zone pressure. These forces also limit print sizes and necessitate cumbersome supporting scaffolds as in traditional VP techniques (*11*, *12*). From the lubrication theory, this Stefan adhesion force scales markedly with part radius

$$F_{\text{Stefan}}=\frac{-3\pi R^{4}\mu U}{2h^{3}}$$ (1)

where *R* is the part radius, μ is the resin viscosity, *U* is the print speed, and *h* is the dead zone thickness. These mass transport limitations, although intensely examined in the literature (*13*–*15*), restrict CLIP and VP from efficiently processing highly filled composite resins and from effectively printing with multimaterials.

In the second UV resin–based AM approach, MJ, an inkjet printhead deposits photocurable resin droplets onto a build surface, readily printing with high spatial control, multimaterials, and highly viscous filled composites (*16*–*18*). However, the disadvantages of MJ include highly anisotropic part properties and very slow printing, making it difficult to scale and arguably mainly for prototyping.

Here, we introduce a new AM method that synthesizes these two established AM approaches by injecting resin through CAD software–designed microfluidic conduits in the part, or “viaducts,” into a continuous liquid interface to supply part production (Fig. 1C). Similar to CLIP, this method uses a highly oxygen-permeable window, an apparatus for which is described in fig. S1, to create a dead zone that, in this instance, provides a destination for resin flow from our high-pressure fluidic injection system (Fig. 1D). We call this 3D printing approach injection CLIP, or iCLIP for short.

## RESULTS AND DISCUSSION

### Injection into a dead zone alleviates suction forces to accelerate printing of 3D geometries

**Fig. 2.**
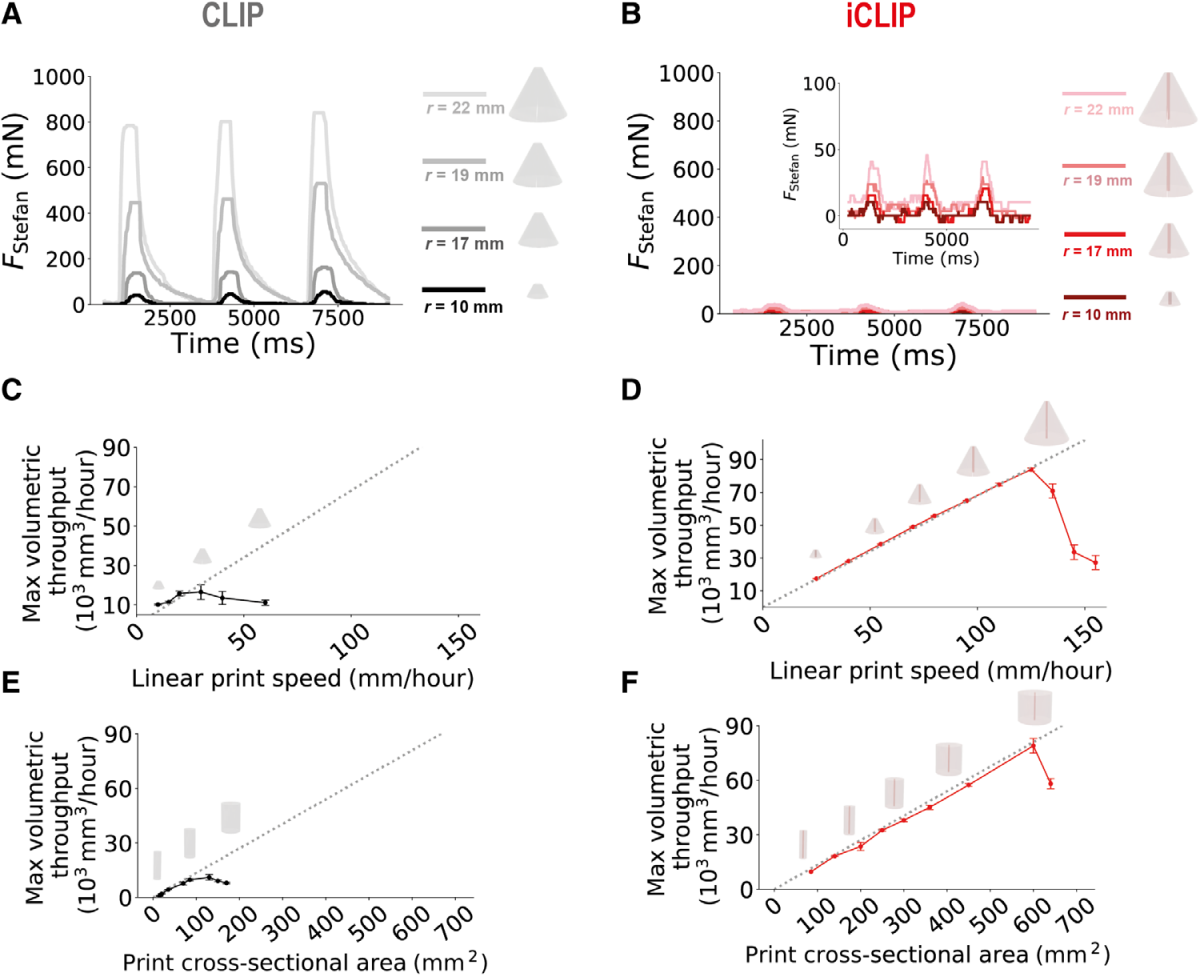
iCLIP accelerates printing of 3D geometries by alleviating suction forces. (**A** and **B**) Experimental load cell force data measured for three consecutive layers, each of 3-s duration, while printing a conical geometry with varying cross-sectional areas by CLIP and iCLIP. (**C** to **F**) Quantified maximum print volumetric throughputs for two test geometries with varying cross-sectional areas, cone (C and D) and cylinder (E and F), by CLIP (left) and iCLIP (right). Gray dotted lines indicate delamination-free prints. Error bars denote ±1 SD from three independent print trials.

Without high-pressure injection, we recapitulate a traditional CLIP process (Fig. 2A), and suction forces scale with part cross-sectional area, as expected from the aforementioned lubrication theory. Without limiting the volumetric print speeds, significant defects and premature delamination from the platform can result. These mass transport limitations can be visualized experimentally using optical coherence tomography (OCT): Without injection, part lifting is accompanied by high-velocity resin influx from part periphery into the dead zone, due to the correspondingly high suction forces (movie S1).

Here, we show that a single viaduct can offset these forces. From the lubrication theory, one can determine that a single viaduct, whose area can be varied dynamically by software during printing, introduces a positive pressure increment by administering resin at a volumetric flow rate *Q*, according to

$$p_{\text{viaduct}}∼\frac{\mu \mathrm{LQ}}{Ah^{3}}$$ (2)

where μ is the resin viscosity, *L* is the part length, *A* is the viaduct area, and *h* is the dead zone thickness. Dead zone thicknesses in iCLIP are very small, as in traditional CLIP and as expected from analytical models (*19*), measured to be on the order of tens of micrometers (fig. S2). With injection, the lubrication theory predicts the (nondimensionalized) pressure field in the dead zone to be

$$\tilde{p}(\tilde{r})=6Q\beta^{2}\text{ln}(\frac{1}{\tilde{r}})+3(\tilde{r^{2}}-1)$$ (3)

where $\tilde{p}$ is the pressure, $\tilde{r}$is the radial position, *Q* is the administered resin flow rate relative to part draw rate, and β is the viaduct radius relative to part radius. According to Eq. 3, the positive pressure increment from injection to offset Stefan adhesion forces is directly proportional to the flow rate administered, along with the relative size of the duct facilitating flow, and decays with distance from the duct. Integrated over the newly cured part surface, we obtain a revised (nondimensionalized) Stefan adhesion force

$${\tilde{F}}_{\text{Stefan}}^{\text{iCLIP}}=\int_{r=0}^{r=1}\int_{\theta =0}^{\theta =2\pi }\tilde{p}(\tilde{r})=\frac{\pi (\beta^{2}Q-3)}{2}$$ (4)

We find that this positive pressure increment allows iCLIP to significantly reduce Stefan adhesion forces—for large area parts by almost two orders of magnitude (Fig. 2B)—and eliminates common defects in CLIP-based printing (fig. S3). While we still observe some scaling of measured Stefan adhesion forces with part cross-sectional area, this scaling is much less marked than in traditional CLIP. OCT visualizations corroborate the reduction in suction forces: In iCLIP, resin flows gradually through the viaduct to supply part production with constant and tunable flow rates (movie S2).

These resin injection ducts enable a significant reduction in suction forces that, in turn, allows iCLIP to achieve significant increases in print speed. For both CLIP and iCLIP, maximum printable speeds for a given cross-sectional area can be quantified as the part draw rate at which delamination events occur, at statistically significant levels, for multiple primitive geometries. Injecting resin through a single central viaduct, we observe increases in the maximum achievable print rates determined to be between 5- and 10-fold (Fig. 2, C to F) over CLIP. For a solid conical part with a cross-sectional radius growing from 2 to 20 mm, for instance, the maximum linear print speed with traditional CLIP before delamination occurs is ~20 mm/hour. With iCLIP, by contrast, maximum print speeds of up to ~125 mm/hour are achievable. With a single resin injection channel, delamination does still occur at an elevated print speed for a given part cross-sectional area due to the difficulties in administering sufficient resin flow through a single duct, a shortcoming that can be overcome by more complex duct geometries as described in the following section.

These ducts need not have detrimental impacts on the mechanical properties of the final part with appropriate postprocessing. In this study, viaduct channels were always sealed after printing by using a postprinting UV cure to the resin-filled channels. When these standard protocols are followed, we do not observe any difference in mechanical properties between CLIP and iCLIP printed dog bones, as shown in fig. S4.

The requirement to integrate ducts into a part can affect the resolution of iCLIP in comparison with CLIP traditionally, but with careful design strategies, this can be minimized. In areas of the part where ducts must be included, feature resolution does drop to the minimum achievable channel diameter before concerns arise such as capillary collapse, if the channel is freestanding, or channel cure through, if the channel is embedded (fig. S5). Moreover, viaduct channels can be designed to have larger radii than this achievable minimum, because of the challenges associated with enforcing viscous flow through a narrow channel, according to the Hagan-Poiseulle equation

$$\Delta P=\frac{8\mu \mathrm{LQ}}{\pi R^{4}}$$ (5)

where *P* is the pressure, μ is the dynamic viscosity, *L* is the channel length, *Q* is the flow rate, and *R* is the channel radius. Flow rates typically range, in this study’s experiments, from 7 to 27 μl/s.

While these channels can therefore be detrimental to iCLIP resolution in some circumstances, note that ducts need not be engineered into all regions of an iCLIP part. Here, the native feature resolution of a traditional CLIP process generalizes to this new printing platform, along with the same trade-off between print speed and feature resolution as characterized by its inventors (*2*). Given that the smallest features are those that experience the smallest Stefan adhesion forces, which thus do not likely require active injection to offset, these high-resolution features can be preserved in iCLIP parts. Moreover, because of the software-guided nature of the integration of ducts into iCLIP parts, ducts can be designed to avoid such high-resolution features.

A second limitation on traditional CLIP is the “material bottleneck,” i.e., filled resins, attractive for their superior mechanical, electrical, and other properties, which are too viscous to flow passively through the thin dead zone. To quantify these limitations, we conducted print studies by traditional CLIP with resins of viscosities ranging from 100 to 7000 cP (fig. S6). If the negative hydrostatic dead zone pressure exceeds cavitation pressure, then dissolved gases nucleate a bubble during printing, manifested as voids in the part (fig. S7, A and B). As expected, these bubbles nucleate at the part’s center, where the negative pressure is predicted to be highest in magnitude. From basic nucleation theory, the negative pressure threshold below which cavitation occurs decreases in magnitude with the temperature of the system *T*, the time elapsed τ, and the saturated vapor pressure of the liquid *P*_sat_, according to (*20*)

$$P_{\text{cavitation}}\propto P_{\text{sat}}-C\;{\left(T\;\text{ln}\;\tau \right)}^{-\frac{1}{2}}$$ (6)

where *C* is a constant. Quantitatively, we observe that this critical cavitation pressure is smaller for more viscous resins, scaling with a predicted minimum negative dead zone pressure of roughly −2 kPa (fig. S7C). Commercial printers can alleviate this problem somewhat by slowing down printing to allow time for resin reflow, by preheating resin in the vat to decrease viscosity, or by performing so-called “pumped” stage motions to encourage reflow, but these do not fundamentally address the mass transport limitations. Combined with the speed limitations described above, a clear and unavoidable trade-off exists between resin viscosity η and printable part radius *r*_p_ in CLIP, according to

$$\text{Speed}\propto \frac{1}{\eta r_{p}^{2}}$$ (7)

**Fig. 3.**
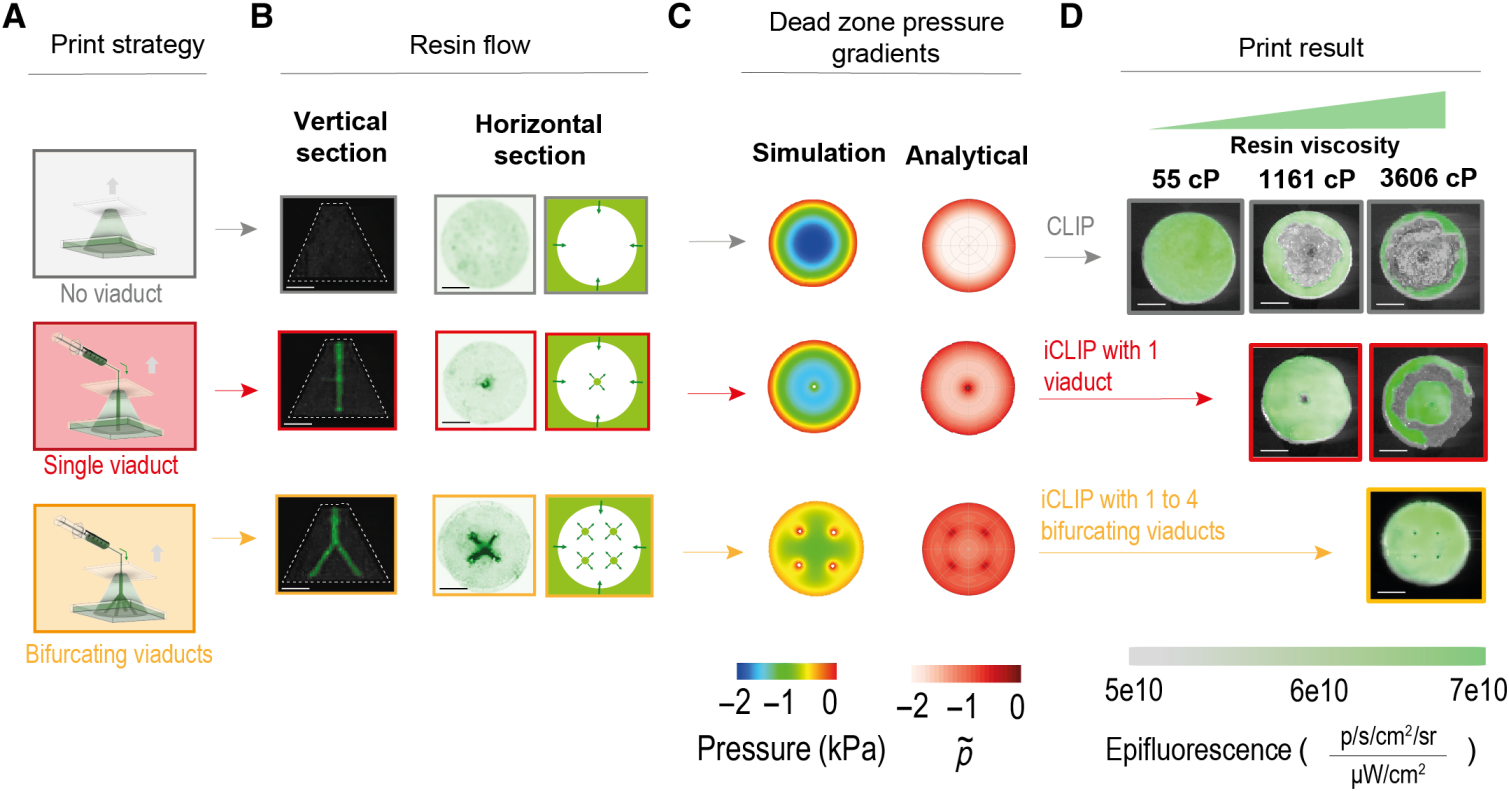
Rapid printing with high-viscosity resins by iCLIP. (**A**) Strategies for printing a cone geometry by CLIP (gray), iCLIP with one viaduct (red), and iCLIP with one-to-four bifurcating viaducts (orange), highlighting resin channels and simulated dead zone pressure gradients. (**B**) Images of iCLIP printed objects with viaducts facilitating resin flow highlighted. (**C**) Pressure gradients within the dead zone predicted by CFD simulation (left) and lubrication theory (right). (**D**) Bottom-up images of CLIP and iCLIP print outcomes with resins of varying viscosities; gray regions indicate cavitation events.

iCLIP circumvents this trade-off by mechanically injecting viscous resin to offset the otherwise negative dead zone pressure, rendering otherwise unprocessable resins printable at higher cross-sectional areas (Fig. 3, A to D). A single viaduct becomes insufficient for extremely high-viscosity resins, as driving viscous flow through a single ~500-μm-radius viaduct causes pump stalling; cavitation recurs between the viaduct source and periphery, which both computational fluid dynamics (CFD) simulations and analytical lubrication theory predict is the region of greatest (magnitude) negative pressure. However, to stay above this critical cavitation pressure, multiple ducts can be engineered into the part, each facilitating positive pressure resin flow into the lubrication theory thin gap. As a result, distributing resin through four bifurcating viaducts once again ameliorates cavitation.

While raising the upper limit on printable resin viscosities in comparison with traditional CLIP, iCLIP is still limited by the high force required to drive viscous flow through a narrow channel, which scales inversely with the fourth power of channel radius at a fixed flow rate according to the aforementioned Hagan-Poiseulle equation (Eq. 5). Even with bifurcating duct arrangements, enforcing flow through narrow microfluidic ducts with resins of viscosities above ~6700 cP became prohibitive, leading to hardware-related pump stalling and subsequent print failure. Still, these viscosities are roughly an order of magnitude higher than those printable by high-throughput traditional CLIP at the same print speeds and part areas. In this manner, iCLIP strikes a balance between high-throughput and high-viscosity 3D printing.

As a result, resins with highly attractive material properties that are too viscous for CLIP are thus accessible to iCLIP, at high volumetric throughputs. Multiwalled carbon nanotubes (MWCNTs), for instance, are extremely attractive additives for their electrical, thermal, and mechanical properties, with applications in, e.g., strain sensors, wearable electronics, and structural health monitoring (*21*, *22*). iCLIP can readily process such a filled resin, forming a body-centered cubic lattice with MWCNT-filled resin flowed through lattice struts at percolation threshold–exceeding concentrations (*23*) without cavitation; as expected, this renders the lattices stiffer but more brittle (fig. S8) (*21*). Resins with up to 1.0 weight % of CNTs were found printable by iCLIP before two factors interfered with reliable printing: the aforementioned challenges associated with driving viscous flow through narrow ducts, along with the decrease in resin penetration depth due to the UV-absorbing properties of CNTs.

Printing with multiple materials simultaneously is key to achieve broad adoption of UV resin–based AM approaches, with potential applications in tunable energy absorption in highly personalized human protection (*24*), wearable electronics (*25*), and functionally graded materials (*26*). Although, with existing VP approaches, multimateriality is only possible using cumbersome vat-switching methods (*27*, *28*), which seriously limits printing speed.

**Fig. 4.**
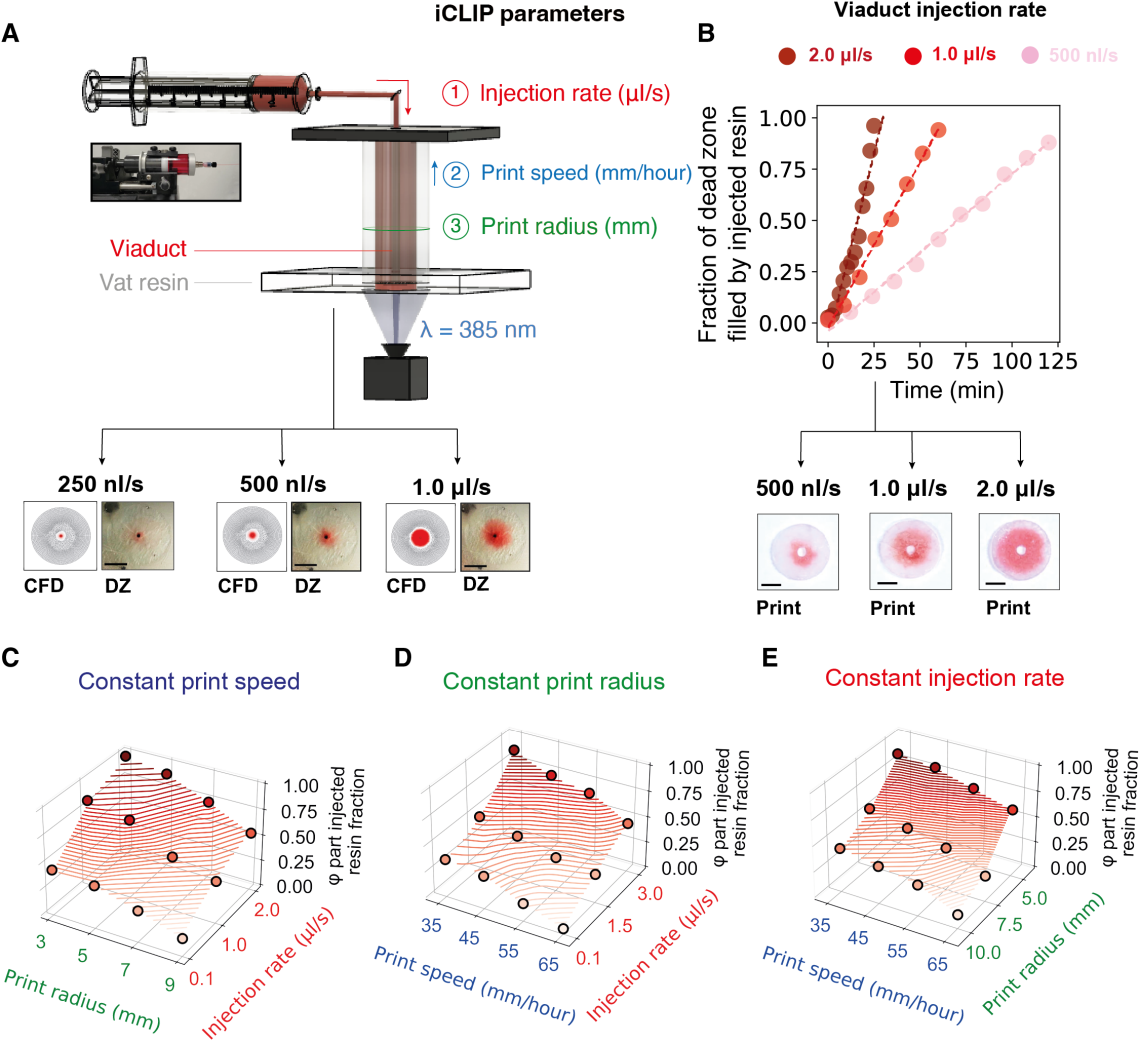
Multimaterial iCLIP control strategy. (**A**) Test geometry for calibrating injection rates during iCLIP, with control parameters that can be tuned during an iCLIP print to adjust the fraction of the vat to injected resin in a part. Below are images of the dead zone during prints with varying injection rates, with corresponding CFD simulation predictions. (**B**) Correlation between administered injection rate and the fraction of the part formed by injected resin, for three different injection profiles. (**C** to **E**) Parameter sweep experiments adjusting one of three control parameters during iCLIP to calibrate material fractions of vat to injected resin in a part. Scale bars, 1 cm. DZ, dead zone.

iCLIP, by mechanically injecting different resins through viaduct(s), can create multimaterial composite architectures in all three Cartesian dimensions. Because no commercially available multimaterial AM design software is available, we implemented a custom CFD simulation–guided and microfluidics-enabled multimaterial control methodology. In a multimaterial iCLIP–printed part, resin may either be supplied by the vat or through injection. Salient parameters for setting this ratio, and thus tuning multimaterial iCLIP printing, include injection rate, print speed, and print area (Fig. 4A). Increasing injection rates lead to a linearly increasing fraction of injected-to-vat resin in the final part (Fig. 4B), as expected, which correlates with higher fractions of the dead zone filled by injected resin (fig. S9). Varying two parameters at a time yielded calibration curves to guide multimaterial iCLIP printing (Fig. 4, C to E). Resin flow can be administered either through ducts fully internal to the part, fully external to the part, or a combination thereof (fig. S10).

**Fig. 5.**
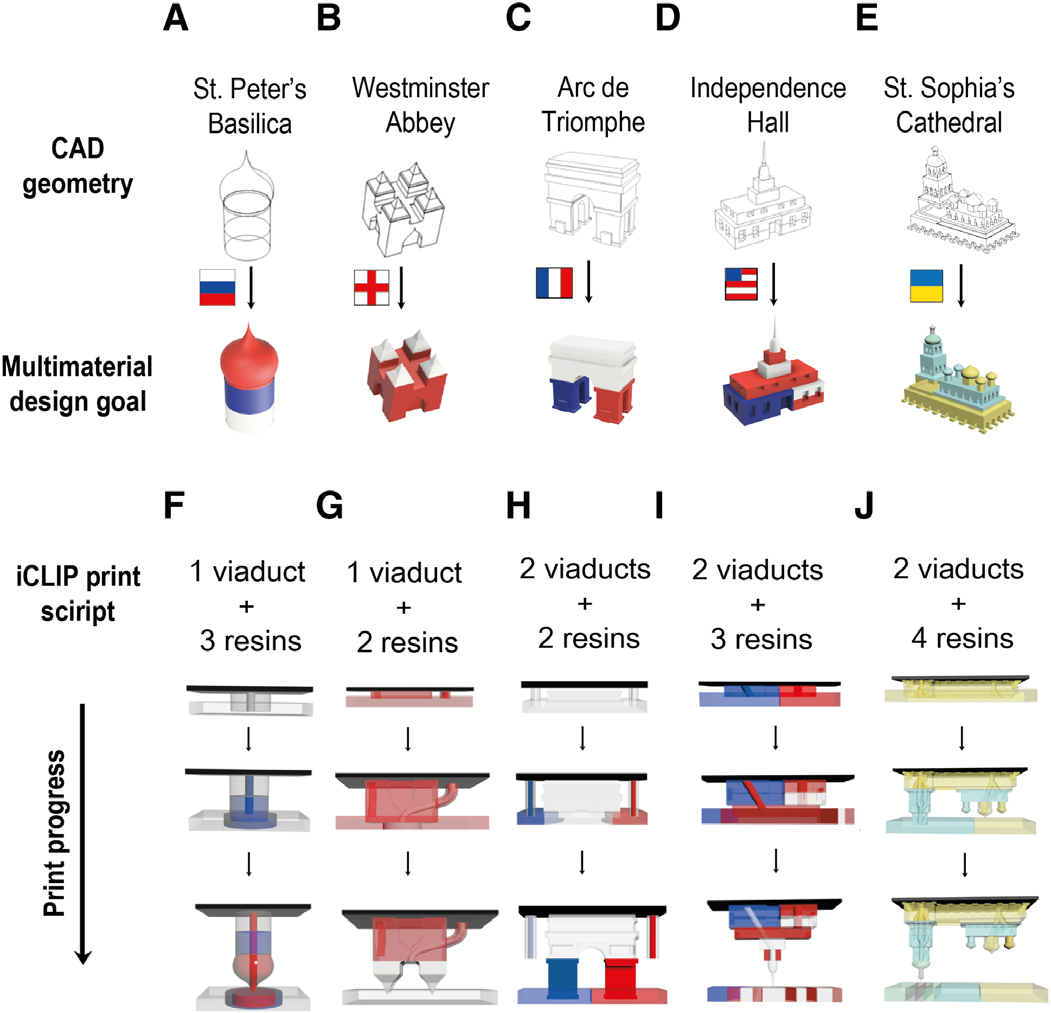
Print scripts for multimaterial iCLIP. (**A** to **E**) Five illustrative multimaterial iCLIP design objectives modeled as historically important buildings (*35*) on which the flag of the country of origin is imprinted in order of increasing complexity. (**F** to **J**) Corresponding iCLIP print strategies highlighting evolving duct geometries over the course of the print. Ducts are engineered internal and/or external to the part to achieve the desired gradients.

**Fig. 6.**
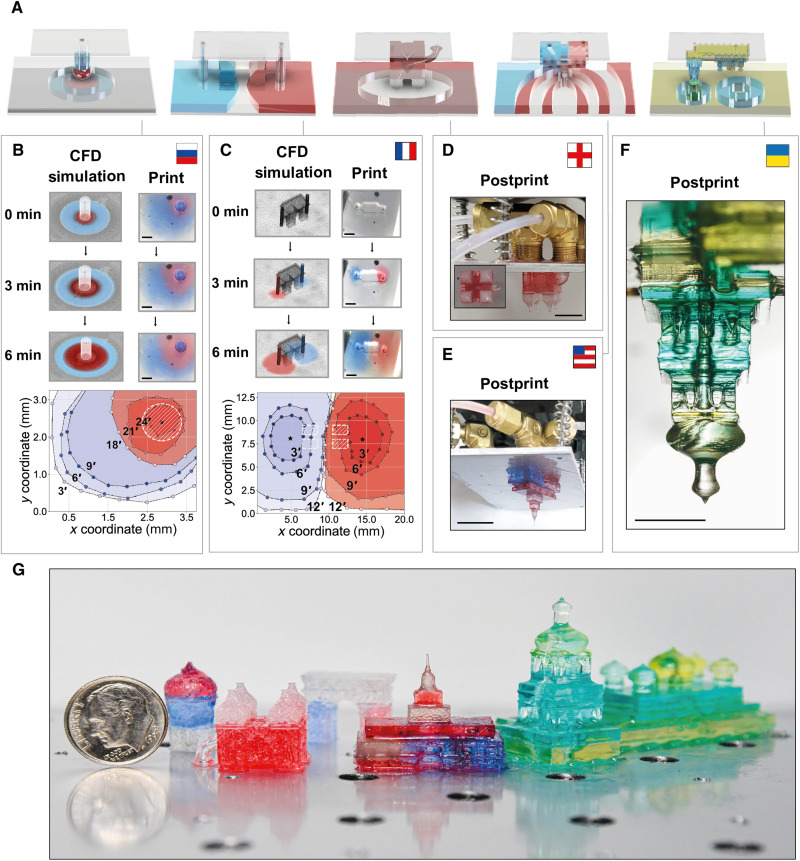
Experimental validation of multimaterial iCLIP print strategies. (**A**) Vat resin distribution goals for multimaterial iCLIP printing flow control strategies. (**B** and **C**) For the St. Basil’s Cathedral and Arc de Triomphe prints, respectively, CFD simulations of flow boundaries induced by injection (left) and images of the resin vat from beneath the window (right), with corresponding digitally extracted flow boundaries at varying time points following the onset of injection (bottom). (**D** to **F**) Multimaterial gradients in Westminster Abbey, Independence Hall, and St. Sophia’s Cathedral prints, and (**G**) all tested models following iCLIP printing. Scale bars, 1 cm.

To demonstrate the feasibility of such a simulation-driven control strategy for more complex designs, we designed injection profiles for five increasingly intricate architectural models, optimizing viaducts and resin flow rates to imprint on each of their country-of-origin flags (Fig. 5). We then validated these print scripts experimentally. Observed injection flow boundaries during printing corresponded with simulation predictions (Fig. 6, A to C), and the resulting models displayed the desired gradients (Fig. 6, D to G).

While not unique in achieving multimaterial 3D printing, compared with existing droplet and extrusion-based multimaterial printing platforms (*29*–*31*), multimaterial iCLIP takes advantage of a continuous liquid interface to achieve high volumetric throughput; specifically, the multimaterial architectures were printed at part draw rates ranging from 50 to 80 mm/hour. Moreover, compared with other multimaterial VP methods that require frequent vat switching by rotating carousels (*32*, *33*), iCLIP minimizes additional hardware accessories required by printing heterogeneous objects in a software-driven manner, engineering ducts into the part in a distinct manner for every print. By administering tunable flow profiles in spatiotemporally controlled fashion, iCLIP also eliminates the need for extensive pauses in printing, as are required by approaches that actively switch out resin every time a material gradient is desired (*28*).

Limitations do, nonetheless, exist on the multimaterial resolution achievable by iCLIP. In particular, the so-called “viscous fingering” during iCLIP printing is viewed from beneath the optically transparent window by a digital imaging camera. In particular, instabilities in the flow boundary between a lower-viscosity resin, injected through a central viaduct into the dead zone, and a higher-viscosity resin, already present in the vat, are readily observed (fig. S11). Nonetheless, it has been shown that viscous fingering can be alleviated by carefully positioning injection points while injecting liquid simultaneously with plate lifting (*34*). Resin contamination during multimaterial iCLIP printing, and thus the potential for added waste, also presents a concern; however, if all injected resin is consumed by part production, as demonstrated by calibration experiments in Fig. 4, then the remaining resin in the vat can be recycled.

**Fig. 7.**
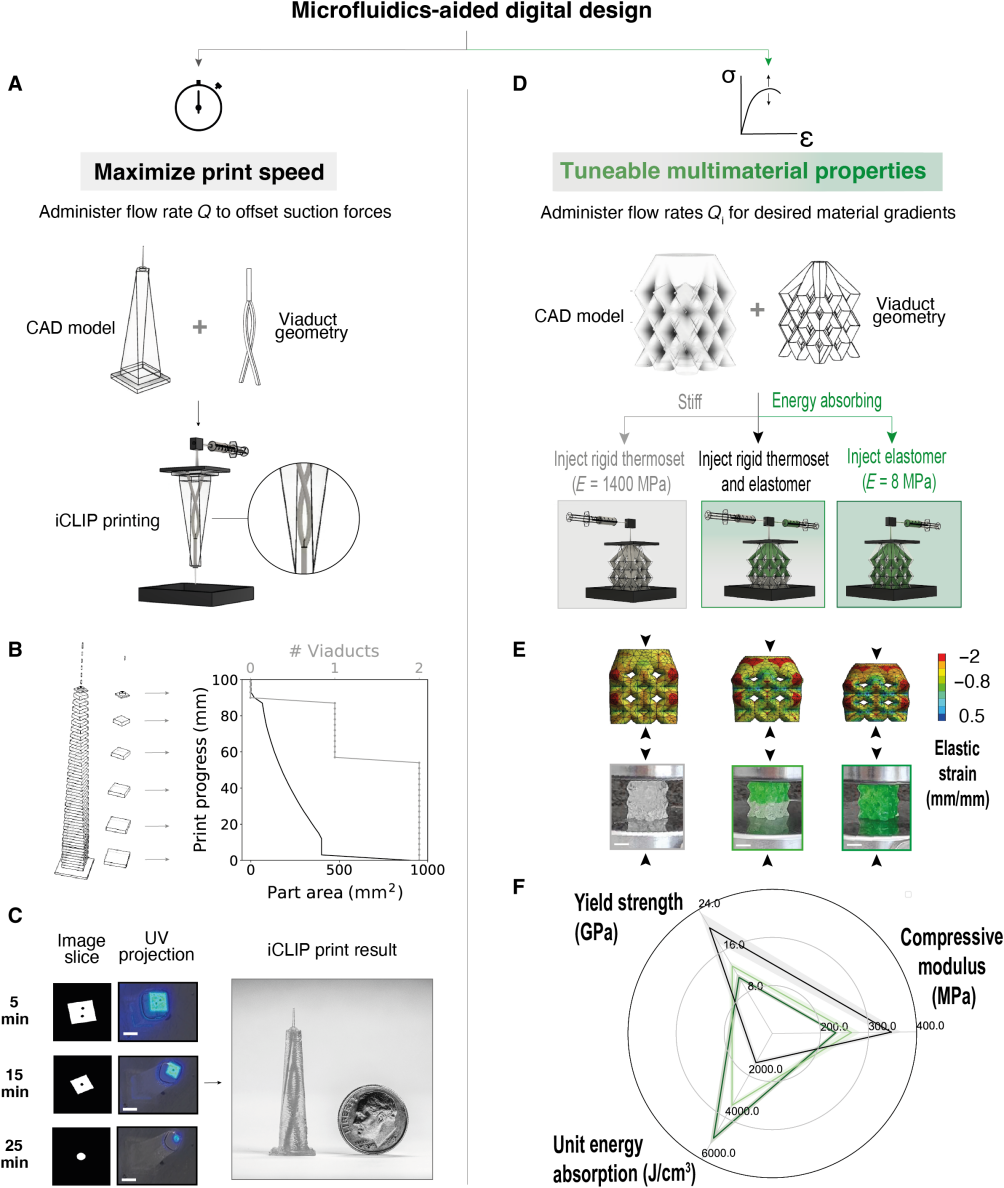
Multiobjective microfluidics-aided digital design for iCLIP. (**A**) To maximize speed, iCLIP parameters are chosen for an input CAD model to minimize mass transport limitations by (**B**) optimizing the number and path of viaducts for changing part cross-sectional area, producing the dynamically changing viaduct path in (**C**). (**D**) To tune part (multi-)material properties, models are infiltrated with viaducts to transport either stiff or elastic resins to the dead zone, guided by (**E**) FEA simulations and experimentally validated by (**F**) mechanical testing in uniaxial compression (rigid lattices in gray, rigid elastomer composite lattices in equal ratios in light green, and elastomer lattices in dark green). Scale bars, 5 mm. Error bars denote ±1 SD from the mean. Simulation deformations are exaggerated for visualization.

The dual print speed and multimaterial goals outlined above require different iCLIP injection profiles, necessitating a new 3D printing design methodology we term microfluidics-aided digital design. To optimize speed, viaducts are digitally designed using a software that implements a dual simulated annealing algorithm and B-spline interpolations to minimize the resin flow distance for a given part cross-sectional area (Fig. 7, A to C). More details of our mass transport optimization algorithm can be found in fig. S12. If tunable multimaterial architectures are desired instead (Fig. 7D), e.g., to modulate a lattice’s energy absorption and/or modulus (Fig. 7E), then viaducts are integrated to alternately transport stiff and elastic resins throughout all lattice struts, thus maximizing control over material distributions. As predicted by finite element analysis (FEA) simulation and confirmed by mechanical testing, increasing injected elastomer volume fractions produced more compliant lattices (Fig. 7F).

In summary, we introduce here iCLIP, a novel 3D printing method using active control of mass transport during continuous liquid interface printing to synergistically enhance print speeds, enable printing of high-viscosity resins, and allow rapid printing of multiple different resins simultaneously at varying scales and with tunable mechanical properties. Ongoing work in optimizing the existing iCLIP process focuses on detailed modeling of the flow boundaries in the dead zone to more finely tune multimaterial gradients, optimizing flow rates to minimize Stefan adhesion forces and cavitation and automating the generation of viaduct geometries and injection profiles to accelerate multimaterial iCLIP printing. Future work in extending iCLIP to new materials and geometries will focus on testing a broader range of viscous-filled resins with superior mechanical and electrical properties for applications in smart and sensor-embedded product designs, along with developing predictive models for analyzing the mechanical properties of multimaterial iCLIP structures for applications in 4D printing and soft robotics, among other areas.

## MATERIALS AND METHODS

### Design of a prototype iCLIP printer

For print platform motion, a Nema 57 stepper motor supplied by a 12-V power bank was used to drive vertical build platform translation along a 30.5 cm Stroke Linear Motion router (VXB Ballbearings, Anaheim, CA, USA). The UV light engine used was a 3DLP9000 (Digital Light Innovations, TX, USA) with a 4 million pixel 2560 × 1600 digital micromirror device (DMD), configured with a 385-nm light-emitting diode (LED) and a 30-μm field-of-view projection lens, with a total projection area of 76.8 mm by 48 mm. The light engine is a combination of a DMD chip set (DLP9000, Texas Instrument, TX) along with a projection lens; the intrinsic specification of the DMD chipset is 385-nm UV wavelength, 2560 × 1600 DMD array, 7.6-μm by 7.6-μm pixel size, and build area of 19.5 mm by 12.2 mm; the projection lens diverges the UV projection to a 2560 × 1600 array of 30-μm by 30-μm pixels to a build an area of 76.8 mm by 48.0 mm at a working distance of 126.5 mm. The printer was coordinated with an Arduino MEGA 2560 microcontroller and RAMPS 1.4 shield running open-source Marlin firmware. Custom software, written in C++ and implemented in the Qt Integrated Development Environment to provide a graphical user interface, allowed for tailoring of UV light intensity, UV exposure time, stage speed and acceleration, layer thickness, and delay time after layers, within and between prints.

### Unfilled resin formulations

For print speed and resin viscosity experiments, resins of tunable viscosity were prepared by mixing isobornyl methacrylate, bisphenol A ethoxylated acrylate, and bisphenol A glycidyl methacrylate at varying ratios with 0.7 weight % of phenylbis(2,4,6-trimethylbenzoyl)phosphine oxide and 0.06 weight % of UV absorber 2-*tert*-butyl-6-(5-chloro-2*H*-benzotriazol-2-yl)-4-methylphenol (BLS 1326), all from Sigma-Aldrich (St. Louis, MO, USA), using a Thinky planetary mixer (Thinky USA Inc., Laguna Hills, CA, USA). Elastomeric resin formulation was prepared with varying ratios of epoxy aliphatic acrylate (trade name Ebecryl 113) and aliphatic urethane–based diacrylate (trade name Ebecryl 8413), which were purchased from Allnex (Malaysia), diluted in isobornyl acrylate and mixed with 1.0 weight % of photoinitiator diphenyl(2,4,6-trimethylbenzoyl)phosphine oxide.

### Filled resin formulations

Carbon nanotube–filled resins were prepared by adding varying amounts of MWCNTs with >95% carbon basis and outer diameter of 20 to 30 nm (CheapTubes Inc. Grafton, VT, USA) to the base polymer matrix and then by subjecting to high shear mixing for 1 min with a planetary centrifuge and ultrasonicating for 2 hours to improve dispersion, periodically replacing the water bath to prevent overheating. To assess dispersion, MWCNT-filled resins were imaged under an Olympus BX53 optical microscope (with UIS2 optical system, infinity-corrected, and Abbe condenser), under 4× objective. Resins were printed immediately after dispersing to preempt MWCNT sedimentation. In addition to their elevated viscosity, making them unprintable by existing CLIP printing approaches, it is well-known that filling photopolymers with MWCNTs, which are UV absorbing and electron scavenging, decreases their cured thickness. To that end, we performed cure thickness experiments as shown in Fig. 2 to adjust UV light intensity accordingly. Specifically, a square five by five grid image was projected at 18 mW/cm^2^ for 30 s. Afterward, the cured thickness at grid locations was measured with a Mitutoyo electronic indicator with precision of 0.5 μm (Mitutoyo American Corp., Aurora, IL).

### Rheological characterization

Rheological characterization as shown in fig. S4 was carried out on uncured (unfilled or filled at different weight percent MWCNT) resin blends using an ARES rheometer [TA Instruments, Sesto San Giovanni (Mi), Italy]. A 25-mm parallel plate configuration was used with 0.1-mm gap between plates. Tests were carried out with the temperature set to 20°C, with shear rate range starting at 1 s^−1^. Viscosities were then determined by the mean apparent viscosity at shear rates between 10 and 30 s^−1^. Apparent viscosity was taken as the average stress/shear ratio between shear rates of 1 and 10 s^−1^.

### Load cell measurements

The build platform was designed to accommodate Miniature S-Beam Jr. Load Cell 2.0 (Futek, Irvine, CA, USA) of dimensions 1.9 cm by 1.75 cm by 0.66 cm, with a resolution of ±0.05%, a rated output of 1 mV/V (250 g) to 2 mV/V (0.453 to 45.3 kg), a bandwidth of 2000 cycles/s, and with signal processing via a USB Load Cell Digital Amplifier (Futek, Irvine, CA, USA). Measurements were taken at 100 Hz. Force data were obtained midway through the print, i.e., once the build platform had fully exited the resin vat such that buoyancy forces did not change between layers.

### Optical coherence tomography

The base unit was the Ganymede Spectral Domain system (GAN621) from Thorlabs (Thorlabs, Newton, NJ, USA), with a center wavelength of 900 nm, a resolution of 3 μm (in air), an imaging depth of 1.9 mm (in air), and an A-scan line rate of 5 to 248 kHz. The GAN621 was equipped with an OCTG9 galvo scanner, and an LK4-BB scan lens with a lateral resolution of 12 μm, 16-mm by 16-mm field of view, and working distance of 42.3 mm. During printing, A-scans were acquired at a frequency of 50 kHz to produce sequential 2D B-scans. Α solid cylinder, either without a viaduct for traditional CLIP or with a viaduct for iCLIP, was printed at ~1 mm from a side of the vat consisting of a transparent glass slide; to capture views from this angle, a rotation mount was used to orient the scanner horizontally. The resin was imaged unfilled, being sufficiently scattering in the near-infrared range to visualize flow.

### CFD simulations

To control multimaterial iCLIP printing, the flow within the dead zone was simulated using commercially available CFD software (ANSYS Fluent, Canonsburg, PA, USA). The resin was simulated as a homogenous fluid with a non-Newtonian viscosity profile. The fluid was given a density of 1120 kg/m^3^. The computational domain consisted of the vat resin and dead zone, modeled as fluid, and the printed object, as solid. The domain was discretized using an unstructured, hexahedral cell mesh composed of a Cartesian core mesh. A no-slip boundary condition was prescribed on the dead zone–window interface. At the viaduct inlet of the computational domain, a mass influx profile is applied corresponding to the prescribed flow rate. The steady-state solution of the flow within the iCLIP printer is obtained by solving the conservation of mass and momentum equations on the computational mesh. Meshes were generated with an element size of 0.01 mm. Simulations were either run as static, when validating against injection calibration experiments, or dynamic, when guiding multimaterial prints.

Simulations were validated experimentally by visualizing the spatial distribution of injected resin in the dead zone, during printing, with red resin dye (non–385-nm UV absorbing) mixed into transparent resin by planetary centrifugation and were digitally imaged from underneath the vat through the optically transparent window. After printing, to quantify the distribution of injected resin in the printed part itself, horizontal ~5-mm cross sections were sliced using a MultiPro Dremel tool and digitally imaged. All image postprocessing and Red-Green-Blue pixel colorimetric quantification and curve fitting were performed in Python using the Python Imaging Library.

### FEA simulations

FEA simulations of three-point bending and uniaxial compression tests on composite prints were run with the commercially available software package ANSYS Mechanical Explicit (ANSYS Inc., Canonsburg, PA, USA), with fixed boundary conditions to simulate outer supports in three-point bending and a downwards pressure condition simulating the load applied.

### Mechanical testing

Before testing, prints were postprocessed by washing with 99% isopropanol, wicking away excess resin with Kimwipes, and post-UV curing by irradiating either in an APM LED UV CUBE II oven (APM Technica, Switzerland, 365 nm) or with a handheld Loctite CureJet UV LED controller (Henkel Corp., Dusseldorf, Germany).

Tensile testing of iCLIP-fabricated ASTM D638 type V dog bones, assessed for dimensional accuracy using a micrometer, was conducted using an Instron 5566 (Universal Testing Systems, Stoneham, MA, USA) with a cross-head speed of 1 mm/min at 25°C to achieve the break at roughly 60 s, which is in accordance to 30 s to 5 min outlined in ASTM D638, with a 100-N load cell. Tensile strength was calculated using the maximum load of the stress/strain curve and Young’s modulus using the linear portion of the stress/strain curve.

Mechanical properties tests of composite prints were performed with an MTS Criterion Model 42 Universal Testing machine (MTS Systems Corporation, Eden Praire, MN, USA), equipped with a 100-N load cell and either with fixtures for three-point bending or platens for uniaxial and transverse compression. For uniaxial cyclic compression tests, the specific energy absorption of the cylindrical specimens (in joules per kilogram) was calculated as the energy dissipated (from the area between the loading and unloading curves in the hysteresis loop) per unit mass. Stiffness (in newtons per millimeter) was measured by subjecting prints to transverse compression at discrete spatial locations in the longitudinal direction and computed as the slope of the load displacement curve. From three-point bending tests, the flexural modulus (*E*) of the cylindrical beams (in newtons per square millimeter) was then calculated as the slope of the flexural stress-strain curve derived from load displacement data as

$$E=\frac{L^{3}}{48I}\frac{F}{\delta }=\frac{L^{3}}{12\pi D^{4}}\frac{F}{\delta }$$

where *I* is the polar moment of inertia for a cylinder under bending, *L* is the crosshead support distances, *D* is sample diameter in millimeters, *F* is load in newtons, and δ is deflection in millimeters.

## Supplementary Materials

### This PDF file includes:

Notes S1 to S9 Figs. S1 to S12 References
