# Supplementary material for: Injection continuous liquid interface production of 3D objects
Source: Sci Adv. 2022 Sep 28;8(39):eabq3917. doi: 10.1126/sciadv.abq3917 (PMC9519045; doi:10.1126/sciadv.abq3917)
Supplement: Supplementary file 1 — Notes S1 to S9 Figs. S1 to S12 References [file sciadv.abq3917_sm.pdf]

Science Advances
AAAS

Supplementary Materials for

Injection continuous liquid interface production of 3D objects

Gabriel Lipkowitz *et al.*

Corresponding author: Joseph M. DeSimone, jmdesimone@stanford.edu

Sci. Adv. **8**, eabq3917 (2022)
DOI: 10.1126/sciadv.abq3917

The PDF file includes:

Notes S1 to S9
Figs. S1 to S12
References

Other Supplementary Material for this manuscript includes the following:

Movies S1 to S3

Supplementary Notes

Supplementary Note 1: Derivation of pressure and velocity fields with and without viaduct flow

To predict the pressure gradients and velocity flow fields in CLIP traditionally, and with injection in iCLIP, because the dead zone gap is in this case very small compared to the part radius, we can use the lubrication equation for nondimensionalized pressure, expressed in polar coordinates:

$$\widetilde{\nabla^2}\tilde{p} = \frac{12}{\widetilde{h^3}}\widetilde{u_z}(\tilde{r},\theta)$$

where $\tilde{r}$ is radial distance, θ angle, and $\widetilde{u_z}$ the vertical velocity field, and $\tilde{h}$ dead zone thickness. This is non-dimensionalized with the characteristic part radii, dead zone thicknesses, pressures, and print velocities:

$$\tilde{r} = \frac{r}{R}$$
$$\tilde{h} = \frac{h}{b}$$
$$\tilde{p} = \frac{p}{\frac{\mu U R^2}{b^3}}$$
$$\tilde{u} = \frac{u_z}{U}$$

where U is part draw rate, R part radius, μ resin viscosity, and b dead zone thickness. Assuming dead zone thickness is constant, i.e.

$$\tilde{h} = 1$$

then:

$$\widetilde{\nabla^2}\tilde{p} = 12\widetilde{u_z}(\tilde{r},\theta)$$

If we assume a constant dead zone thickness, reducing $\tilde{h}$ to 1, this results in a negative pressure $\widetilde{p^{(1)}}$ in the dead zone without injection due to upwards part drawing:

$$\tilde{p}^{(1)} = 3(\tilde{r}^2 - 1)$$

Integrated over the part's cross-sectional area, this yields the familiar Stefan force, which nondimensionalized is:

$$\tilde{F}_{Stefan} = \frac{-3\pi}{2}$$

The negative quantity indicates the force is acting downwards, "pulling" against the adhesion of the part to the platform. Expressed in dimensional form, this is:

$$F_{Stefan} = \frac{-3\pi\mu R^4}{2b^3}$$

The radial velocity field can then be simply derived as, without viaduct flow:

$$u_r = -6\tilde{r}z(1-z)$$

where z is the vertical distance above the window in the dead zone. Again, using the lubrication theory equation for pressure to determine the pressure increment from viaduct flow, where Q is defined as the flow velocity out of the viaduct relative to the part draw rate U, the pressure increment due to viaduct injection is:

$$\widetilde{\nabla^2}\tilde{p} = -\frac{12Q}{\widetilde{h^3}}$$

Evidence that this physical model, also known as a lifting plate Hele-Shaw gap *(36)*, indeed accurately describes the iCLIP process comes from observations of viscous fingering *(37)* during iCLIP printing, as viewed from beneath the optically transparent window by a digital imaging camera. In particular, instabilities in the flow boundary between a lower viscosity resin, injected through a central viaduct into the dead zone, and a higher viscosity resin, already present in the vat, are readily observed (**Figure S11**).

Within the viaduct, i.e., for $\tilde{r} \leq \beta$, this results in a combined pressure of:

$$\tilde{p} = \widetilde{p^{(1)}} + \widetilde{p^{(2)}} = 3\left(\widetilde{r^2} - 1\right) + 3Q\left(\beta^2 - \widetilde{r^2}\right) + 6Q\beta^2 ln\left(\frac{1}{\beta}\right)$$

And for $\tilde{r} > \beta$:

$$\tilde{p} = 3\left(\widetilde{r^2} - 1\right) + 6Q\beta^2 ln\left(\frac{1}{\tilde{r}}\right)$$

which is clearly continuous at $\tilde{r} = \beta$ and well-defined everywhere. Integrating over the surface of the cured part, i.e., outside the viaduct at $\tilde{r} > \beta$, this yields a revised (again nondimensionalized) Stefan force:

$$F_{Stefan} = 3\pi Q\beta^2 - \frac{3\pi}{2}$$

For the revised velocity fields with viaduct flow, specifically within the viaduct i.e., $\tilde{r} \leq \beta$, we derive:

$$u_r = \left(-6\tilde{r} - 3Q(2\tilde{r})\right)z(1-z)$$

And outside the viaduct, i.e., $\tilde{r} > \beta$:

$$u_r = \left(-6\tilde{r} + 6Q\beta^2\left(\widetilde{r^{-1}}\right)\right)z(1-z)$$

Supplementary Note 2: Delamination print failure mode experiments

The limitations on CLIP and iCLIP print speeds were quantified by printing one of two test geometries, a cylinder or spherically symmetrical cone, according to the print scripts in **Figure S3a** and **Figure S3e**, respectively. Prior to each print, resin in the vat was stirred to ensure homogeneity and filled to the same height in the vat. Only one part was printed per experimental trial so that resin flow from adjacent parts would not interfere with the fluid dynamics governing flow for one part. Overall print speed was varied by systematically changing the time between UV exposures ("delay time"), and correspondingly the stage velocity. All other parameters (e.g., UV light intensities, resin formulations, exposure times, printer hardware) were held constant, to isolate injection as the sole difference between traditional CLIP and iCLIP trials. Thanks to the geometrical versatility of our software-driven resin delivery approach, iCLIP prints could be conducted with a wide range of viaduct cross sectional areas, from 5 to 50% of part area, which could be dynamically tuned between, or over the course of, single prints. Trials for iCLIP and CLIP were performed in alternated fashion, to further control for any extraneous external factors (e.g., changing resin temperature, oxygenation conditions, etc.). Then, to quantify print failures in print speed experiments, the cross-sectional area at which delamination occurred was measured with a stainless-steel digital Vernier caliper, with an accuracy of 10 μm. The maximum printing speed for a given cross sectional area was quantified as the overall speed at which average print height dropped below the design print height at statistically significant levels ($p < 0.05$), using a two-way ANOVA test of statistical significance.

Supplementary Note 3: Optical Coherence Tomography imaging of mass transport in CLIP and iCLIP

The suction forces limiting the traditional CLIP process can be visualized using optical coherence tomography. In CLIP, the continued mobility of particles even after UV exposure provides evidence of a dead zone near the window interface. From a Lagrangian fluid dynamics perspective, which treats an observed area of near-IR scatter as a surrogate marker for resin particles, fluid accelerates in the dead zone from near quiescence to velocities approaching ~10^3 μm/s during part lifting (**Movie S1**). At volumetric injection rates of 1 μL/s and 10 μL/s, fluid velocities of ~10^2 μm/s and between 10^3 and 10^4 μm/s are observed, respectively. From recordings of flow through the dead zone in traditional CLIP, the large magnitude pressure gradients are reflected in the very high fluid particle accelerations observed (**Movie S1**). Notably, particle motion into the dead zone is only observed simultaneous with platform lifting, i.e., due to suction forces. In contrast, while injecting resin at a constant rate during printing, continuous flow in the opposite direction of part movement can be observed (**Movie S2**). As shown in the video, such flow rates can be tuned to be either lower or higher than part production. In either case, the absence of dramatic fluid particle accelerations, i.e., the decoupling of part lifting with mass transport, provides visual evidence for the lower suction forces quantified in **Main Text Figure 2**. Here, the absence of high fluid particle accelerations provides visual evidence of the alleviation of suction forces, demonstrating how increasing flow rates can offset increasing suction forces.

Supplementary Note 4: Cavitation print failure mode experiments

For quantifying the resin viscosity limitations of the traditional CLIP process, viscosity was varied by changing the percentage of high viscosity bisphenol A glycerolate methacrylate vs. low viscosity bisphenol ethoxylate diacrylate in the formulation (**Figure S6a**). The viscosity range of these custom resins was comprehensive (**Figure S6b**), with the minimum below and the maximum exceeding those of commercial CLIP and SLA resins (**Figure S6c**). When printing with these resins, the cross-sectional area at which cavitation occurred during printing was then captured by imaging the print from underneath the vat (**Figure S7**). To visualize cavitation print failure results, fluorescent resin dye (Alumilite, Galesburg, MI, USA) was pipetted into the cavitation region after printing and imaged using an In Vivo Imaging System (IVIS) 100 Series, with a Cy5.5 excitation filter of 430 nm and ICG emission filter 500 nm (PerkinElmer, Waltham MA, USA). In all fluorescence images to visualise the cavitation process, excitation and emission filters, and maximum captured epi-fluorescence (radiant efficiency), and fluorescent dye concentration, were kept constant.

The cavitation failures observed with these resins due to the evaporation of dissolved gases in viscous resins have been noted by others, specifically those performing continuous printing with a liquid window noting that for large cross-sectional areas, such large negative pressure gradients in fact lift the liquid window material into the part, causing cavities *(41)*. While ours is a solid, stiff window, we observe similar cavitation failures in parts, as shown in **Figure S7**.

Cavitation failures are not a limitation of only CLIP, but also of all light-based resin printing; in traditional SLA, the lift mechanism can, if the part's cross-sectional area is large enough, leave an empty area in the vat. Strategies do exist to alleviate this in traditional SLA, e.g. with wipers. In the CLIP process, the lift mechanism is eliminated, but time is still required to allow resin to reflow through the dead zone, which can be even longer due to its thin height and the unfeasibility of any wiping mechanism. Commercial printers attempt to alleviate this problem by heating the resin vat to accelerate reflow, or utilising "pumping" mechanisms whereby the stage motion can facilitate reflow, but this does not accelerate resin reflow dramatically, as mechanical injection in iCLIP can.

Supplementary Note 5: Negligible impact of post-cured viaduct on iCLIP printed part mechanical properties.

If left uncured, a concern with iCLIP is that hollow viaducts would diminish the mechanical properties of printed parts. To ameliorate this issue, we rendered our parts fully solid by post UV curing resin left in the viaducts. To demonstrate equivalent mechanical properties to traditional CLIP printed parts, we printed ASTM D638 Type V dogbones both via traditional and iCLIP (**Figure S4a**), thereafter washing with isopropyl alcohol and post UV curing the resin-filled viaducts in a APM LED UV CUBE II (APM Technica, 365 nm) for 2 minutes. As a negative control, we pipetted out the resin from the viaducts of a second group of iCLIP printed dogbones, leaving behind a hollow channel (**Figure S4b**). All samples were dimensionally accurate, satisfying ASTM specifications (**Figure S4c**). Then, tensile tests were performed on these specimens. While a statistically significant decrease in ultimate tensile strength (UTS) and Young's modulus was observed for viaduct-uncured dogbones, no such difference was observed when the viaduct was post-cured (**Figure S4d-e**). This demonstrates that with proper post-processing, iCLIP viaducts that are embedded within the part itself need not affect part mechanical properties.

Supplementary Note 6: Optimization of viaduct geometry during iCLIP

To achieve flow through viaducts to the dead zone, two defects in viaduct geometry are common and must be avoided: lateral cure through due to too high UV intensity, and unvented cavities due to insufficient resin flow, as explained below.

The problem of vertical cure through is well-studied in the 3D printing literature *(38-40)*, but for iCLIP, lateral cure through is also a concern (**Figure S5**). Namely, the integrity of the viaduct is crucial to maintain during iCLIP; if obstructed by lateral cure through due to light piping or "funneling", whereby an optically transparent material (such as an acrylate) directs light through a low refractive index material, then injected resin exacerbates, rather than alleviates, the aforementioned suction forces pulling down on the part, and also causes random macroscopic channels and voids in the part from uncontrolled fluid flow escape (**Figure S5c-e**). Preventing this is possible, however, by calibrating the projected UV light intensity to the desired software-designed viaduct size (**Figure S5f**). To demonstrate, parts were printed to the equivalent of 100 layers, washed with isopropanol, and then channels measured with an Olympus-BX53 optical microscope (with UIS2 optical system, infinity-corrected, and Abbe condenser), under 4x objective. With such calibration, viaduct radii of 500 μm are readily achievable in UV intensity ranges sufficient for printing (**Figure S5g**).

Conversely, viaduct diameter may expand uncontrollably without sufficient injected resin flow. The physical reason is the well-known design limitation of unvented cavities in resin printing, which prohibits geometries with voids lacking ventilation. While some have creatively exploited the physics of unvented cavities in CLIP to draw uncured resin into voids in the part and make thermoset-elastomer composite structures *(41)*, we find that when no resin is injected into a central viaduct, the print “bursts” from the low pressure build-up in such unvented cavities.

Supplementary Note 7: Optimization of viaduct position and flow rates during iCLIP

Determining the optimal position of and flow rate through viaducts during iCLIP is key to achieving its technical improvements over the traditional CLIP process. In addition to allowing higher print speeds by alleviating suction forces, as shown in **Main Text Figure 2**, and alleviating cavitation, as shown in **Main Text Figure 3**, we also find that injection may help to prevent dead zone thicknesses from shrinking towards the center of the part where fresh resin from the periphery struggles to reflow (**Figure S2d-e**), as others have observed *(41)*.

The process for finding the optimal viaduct placement for an image slice for a given layer, as shown in **Figure S12**, is as follows. The image slice and initial guess for viaduct placement coordinates is fed into a dual simulated annealing optimization loop which outputs optimized viaduct placement coordinates. The optimal viaduct number and placement is determined by quantifying the distance from the part edge to the deepest pixel, for increments during the print. The output is, for each image slice in the geometry, a sequence of optimal resin source positions that can be interpolated smoothly with B splines in standard CAD software to reconstruct a revised CAD geometry with viaducts for resin flow.

The calibration of injection rates through viaducts differs based upon the goal of iCLIP: if multi-material printing is desired, the flow boundaries must be monitored according to **Main Text Figures 4**, along with **Figures S9-10**. If seeking to alleviate suction forces and negative hydrostatic pressures in the dead zone gap causing either cavitation or delamination failure, instead, an injection rate that completely satisfies part production can be administered, for a simple cylinder, according to:

$$Q=U\pi r^2$$

where Q is flow rate, U is continuous print speed, and r is part cross-sectional radius. Flow rates typically range, in this study's experiments, from 7 μL to 27 μL.

Theoretically, a single viaduct facilitating pressure driven resin flow could offset Stefan adhesion forces and/or the negative dead zone pressure over any arbitrary area or viscosity by correspondingly increasing flow rate. However, when resin viscosity η and/or print cross-sectional radius r grow high enough, it becomes unfeasible to infuse this volumetric flow rate through one 500 μm radius viaduct port, which produces too high a backpressure for the syringe pump to overcome, causing pump stalling. Quantitatively, the force required to pump an incompressible, Newtonian fluid of arbitrary viscosity experiencing laminar flow with low Reynolds number through a pipe, which accurately describes resin flow through a viaduct, scales according to the Hagan Poiseuille law with $\frac{\eta}{r^4}$ where η is resin viscosity and r is the viaduct radius. According to CFD simulations, creating a net zero pressure across the cross-sectional area of the part with high viscosity resins necessitates a very high positive pressure at the viaduct port, causing pump stalling. For a resin of viscosity 3600 cPs, for instance, this was empirically determined to be a part radius of 14.6 mm.

As such, it becomes more practical to distribute resin flow through several viaducts, which bifurcate through the z axis, to offset the negative pressure in the dead zone with a more uniform hydrostatic pressure distribution across the part's cross-sectional area, as shown in **Main Text Figure 3**.

Supplementary Note 8: Control methodology for multi-material iCLIP printing

A flow matrix system can then be used to dynamically toggle the resin injected through a particular viaduct during printing to produce the gradients modelled in **Main Text Figure 5**. For St. Basil's Cathedral, a single viaduct conduits three different resins to the dead zone to produce z axis gradients in a single print, i.e., the Russian tricolor. For the Arc de Triomphe, two viaducts transport red and blue resins, along with existing white vat resin, to produce the French tricolor. For Westminster Abbey, resin is flowed first in a viaduct external to the object and then merged into the part's interior, to produce the cross of St. George viewed from below. And for Independence Hall and St. Sophia's Cathedral, resin is flowed through multiple viaducts internal to the object to obtain gradients in both the horizontal and vertical directions, to produce the American and Ukranian flags, respectively. By guiding the flow boundaries according to the CFD simulation predictions for iCLIP shown in e.g. **Main Text Figure 4**, the multi-material prints shown in **Main Text Figure 6** are achieved.

If minimal part disruption is desired during such multi-material iCLIP printing, the viaduct need not be fully internal to the part. For this approach, a support viaduct begins external to the part, thereafter becoming internalized (**Figure S10c**). An added benefit is that, like the flying buttresses that provide structural strength to the walls of Westminster Abbey, external viaducts act as pseudo-support structures during the print. During iCLIP printing, thanks to the moving software-integrated dark spot in the UV projection (**Figure S10d**), injected resin can flow through the external viaduct and to the part build area, producing the desired Cross of St. George pattern in the print (**Figure S10e**).

Supplementary Note 9: Resolution-throughput tradeoff in iCLIP compared with other multi-material printing approaches.

From print hardware parameters, e.g, nozzle diameter, linear feed rate, and material switching times in direct ink write, inkjet, and polyjet 3D printers, it is possible to estimate resolution and throughput. While not taking into account factors such as “slump” in FDM printers extruding viscoelastic material, or start up and post-processing times, these are useful for comparative purposes. Of the reported multi-material polymeric 3D printing approaches, fused filament fabrication (FFF) can have very high throughput of multiple different extruded thermoplastics, but resolution through such large nozzles sizes is limited *(42-47)*. Selective laser sintering (SLS) with multiple powders, while higher in resolution than FFF and benefitting from high laser scan rates, still suffers from the resolution limitations of the resulting melt *(48-50)*, Direct ink write (DIW), and especially inkjet (IJ), approaches can display even higher spatial resolution, but volumetric injection rates are low, leading to low throughput *(51-56)*. And traditional vat photopolymerization (VP) approaches, which can be high resolution as well, nonetheless suffer in throughput from the requirement to lift and retract every layer *(57-58)*.

Unlike these multi-material printing platforms, iCLIP combines controlled mass transport of multiple materials in a high throughput printing process, with high pixel resolution. iCLIP, with a 30 μm UV projection pixel resolution and 50 mm/hr print speed implemented during multi-material print experiments, does not exceed the throughput of the fastest FFF multi-material printers or the resolution of the most detailed IJ printers, but strikes a unique balance between these considerations.

Supplementary Videos

Movie S1: Optical coherence tomography visualization of suction forces during traditional CLIP printing. Cross sectional view of a growing cylindrical part during the traditional CLIP process, implemented by a stepped printing motion as can be observed by the stepped motion of particles. The oxygen permeable window is on bottom and the growing part on top. Inset view is of the continuous liquid interface, with rapid fluid flow due to suction through this dead zone, towards the part center. U is the part draw rate, in mm/hr. Scale bar denotes 100 μm.

Movie S2: Fluid flows during traditional CLIP and during iCLIP at varying volumetric flow rates. Cross sectional view of fluid flow surrounding a growing cylindrical part during traditional CLIP or iCLIP. To left, fluid flow towards the dead zone during CLIP, and to right, through iCLIP viaducts at volumetric flow rates either below or above that required for part production (left and right, respectively). U is the part draw rate, in mm/hr, Q is the viaduct flow rate, in μL/s, r is the part radius, in mm, and t is time. Scale bars denote 100 μm. Grey outlines the growing part, as evidenced by the observed stepped motion of particles during the stepped print script.

Movie S3: Multi-material iCLIP print strategy. For achieving a two-material iCLIP print, flow rates are changed dynamically and continuously during printing, without having to pause as in traditional SLA-based multi-material printing. Note that this is only one of many possible mechanisms for achieving the multi-material pattern. Flow rates vary from 27 μL/s for the highest cross sectional areas, at the tower base, to 500 nL/s, at the spires.

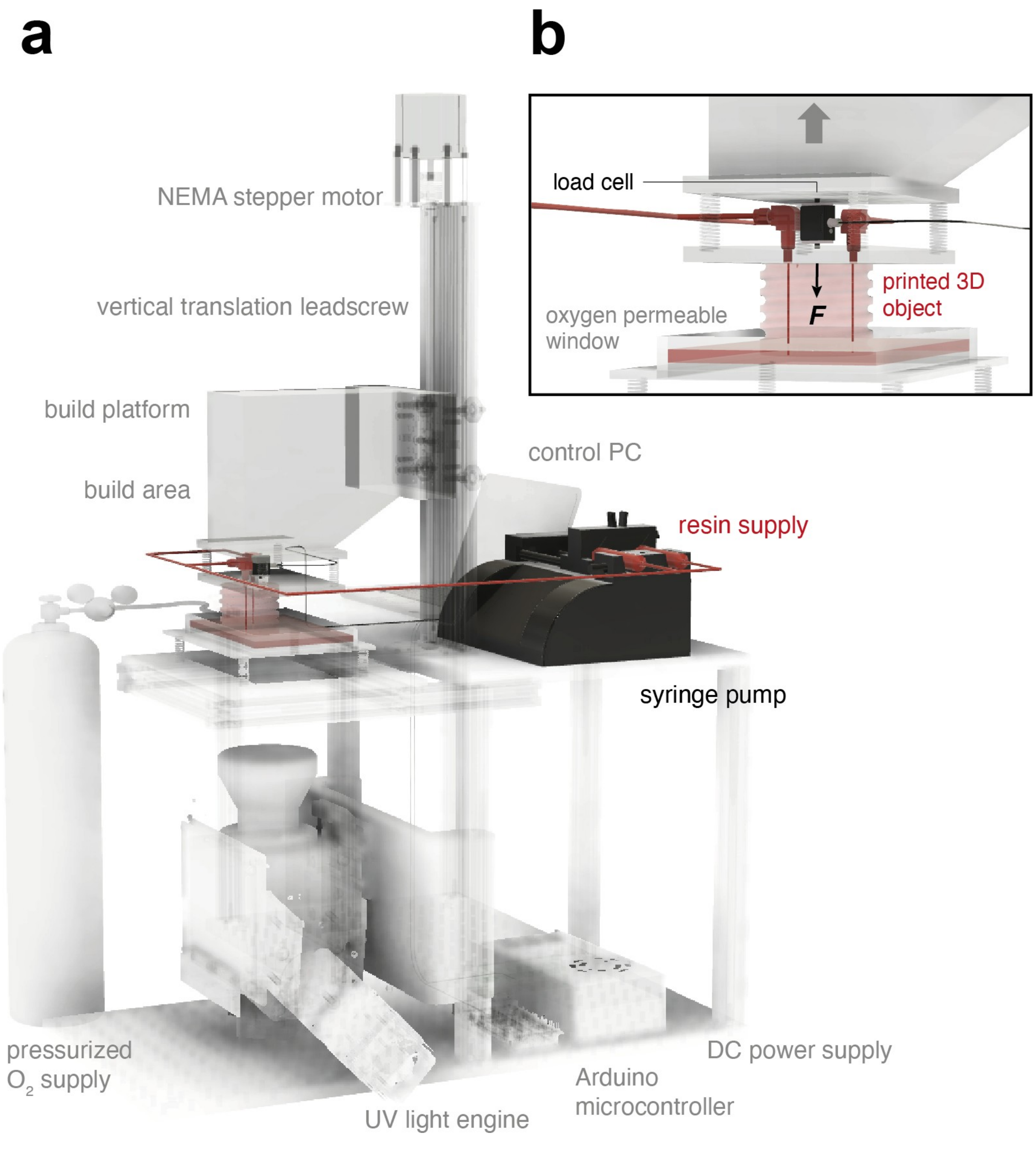

Figure S1: Design of a prototype iCLIP printer. (a) Overall hardware configuration of the iCLIP printer, with salient features of the iCLIP system highlighted. **(b)** The build region of the iCLIP printer, with the mounting of the load cell and fluidic connectivity ports indicated. The downw ards Stefan adhesion force measured by the load cell is indicated by the **black** arrow, while the platform movement is indicated in **grey**.

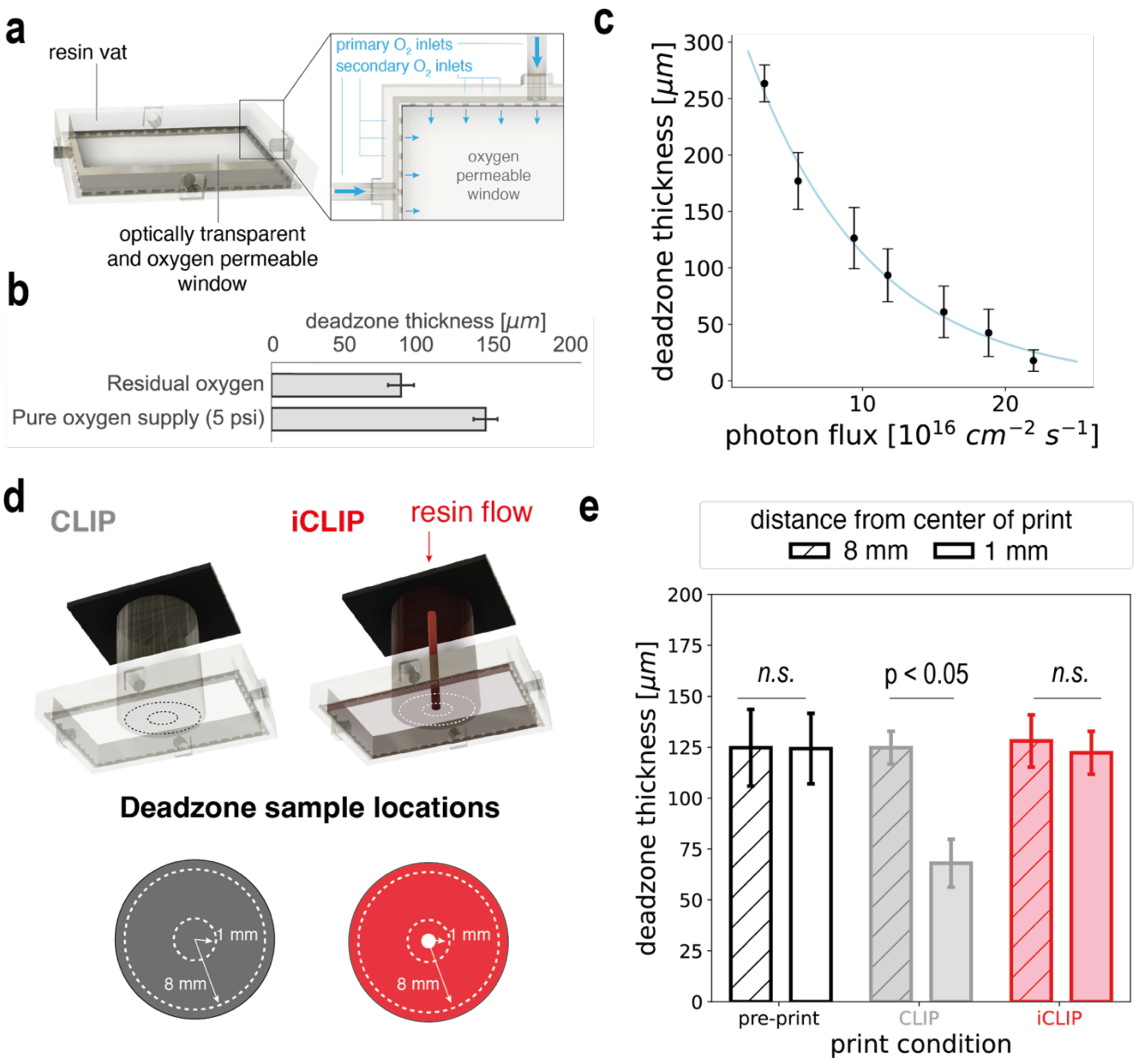

Figure S2: iCLIP depends upon an oxygen-inhibited deadzone as a destination for injected resin during printing. (a) Schematic illustration of the oxygen-permeable window set-up for the prototype iCLIP printer, indicating primary and secondary oxygen inlets that feed the highly oxygen-permeable window. **(b)** Deadzone thicknesses measured with or without active supply of pressurized pure oxygen. **(c)** Experimentally-measured deadzone thicknesses for the iCLIP window at varying UV exposures. **(d)** Experimental method for measuring deadzone thicknesses across the area of a part during CLIP (left, **grey**) and iCLIP (right, **red**). **(e)** Experimentally-measured deadzone thicknesses before printing (left, **black**), after printing by CLIP (middle, **grey**), and after printing by iCLIP (right, **red**).

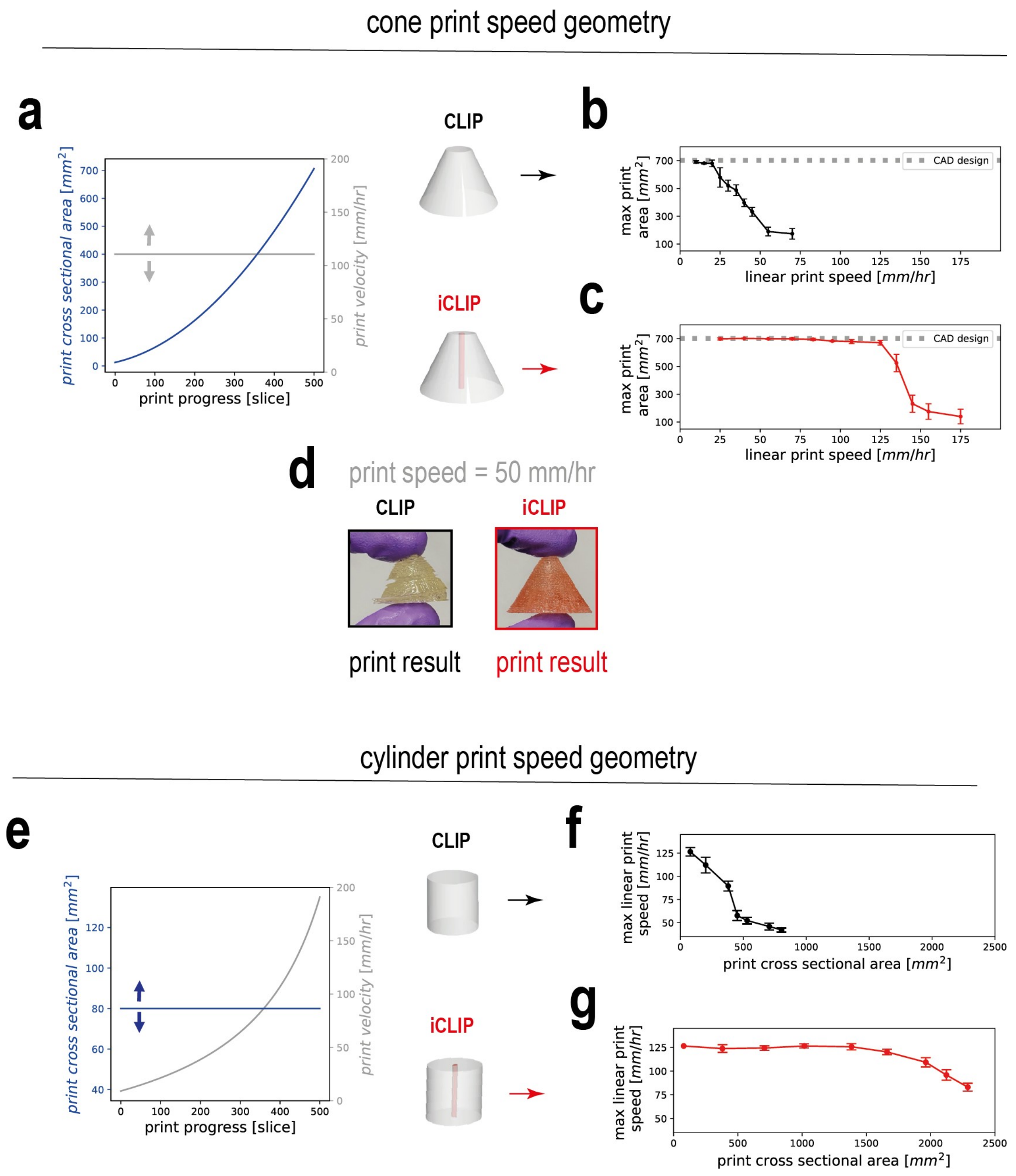

Figure S3: Geometries used to quantify maximum printable speeds of CLIP and iCLIP. (a) Test geometry 1 (cone) and associated print parameters to evaluate the relationship between printable speed and area in traditional CLIP (top) and iCLIP (bottom). **(b-c)** Print failure mode experiments to determine maximum print speed achievable, for the geometry in (a), by CLIP (**black**) and iCLIP with a central viaduct (**red**). **(d)** Images of print defects observed due to Stefan adhesion forces while printing a cone geometry by traditional CLIP (left) not observed under the same printing conditions except with injection in iCLIP (right). **(e)** Test geometry 2 (cylinder) and associated print parameters to evaluate the relationship between printable speed and area in traditional CLIP (top) and iCLIP (bottom). **(f-g)** Print failure mode experiments to determine maximum print area achievable, for the geometry in (d), by CLIP (**black**) and iCLIP with a central viaduct (**red**).

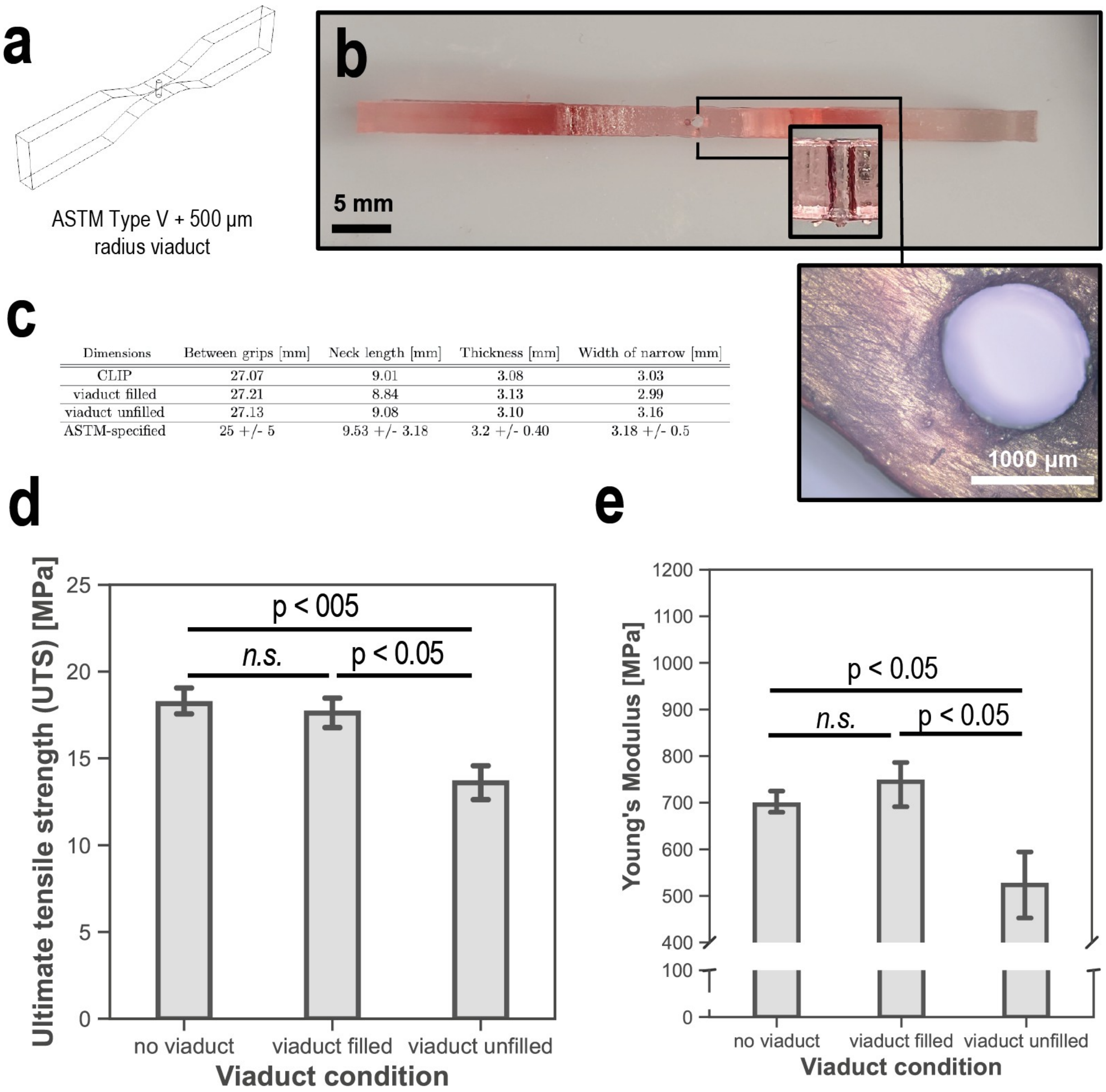

Dimensions	Between grips [mm]	Neck length [mm]	Thickness [mm]	Width of narrow [mm]
CLIP	27.07	9.01	3.08	3.03
viaduct filled	27.21	8.84	3.13	2.99
viaduct unfilled	27.13	9.08	3.10	3.16
ASTM-specified	25 +/- 5	9.53 +/- 3.18	3.2 +/- 0.40	3.18 +/- 0.5

Figure S4: Negligible impact of post-cured viaduct on iCLIP printed part mechanical properties. (a) CAD design of test geometry for evaluating effect of viaduct on dogbone mechanical properties. **(b)** Picture of iCLIP printed dogbone, with inset showing optical microscopy image of central viaduct. **(c)** Comparison of iCLIP printed dogbone with ASTM Type V specifications. **(d-e)** Ultimate tensile strength and Young's modulus, respectively, of iCLIP printed dogbones under different viaduct post-treatment conditions. All measurements represent the average of three independently printed dogbones, with error bars denoting +/- one standard deviation from the mean.

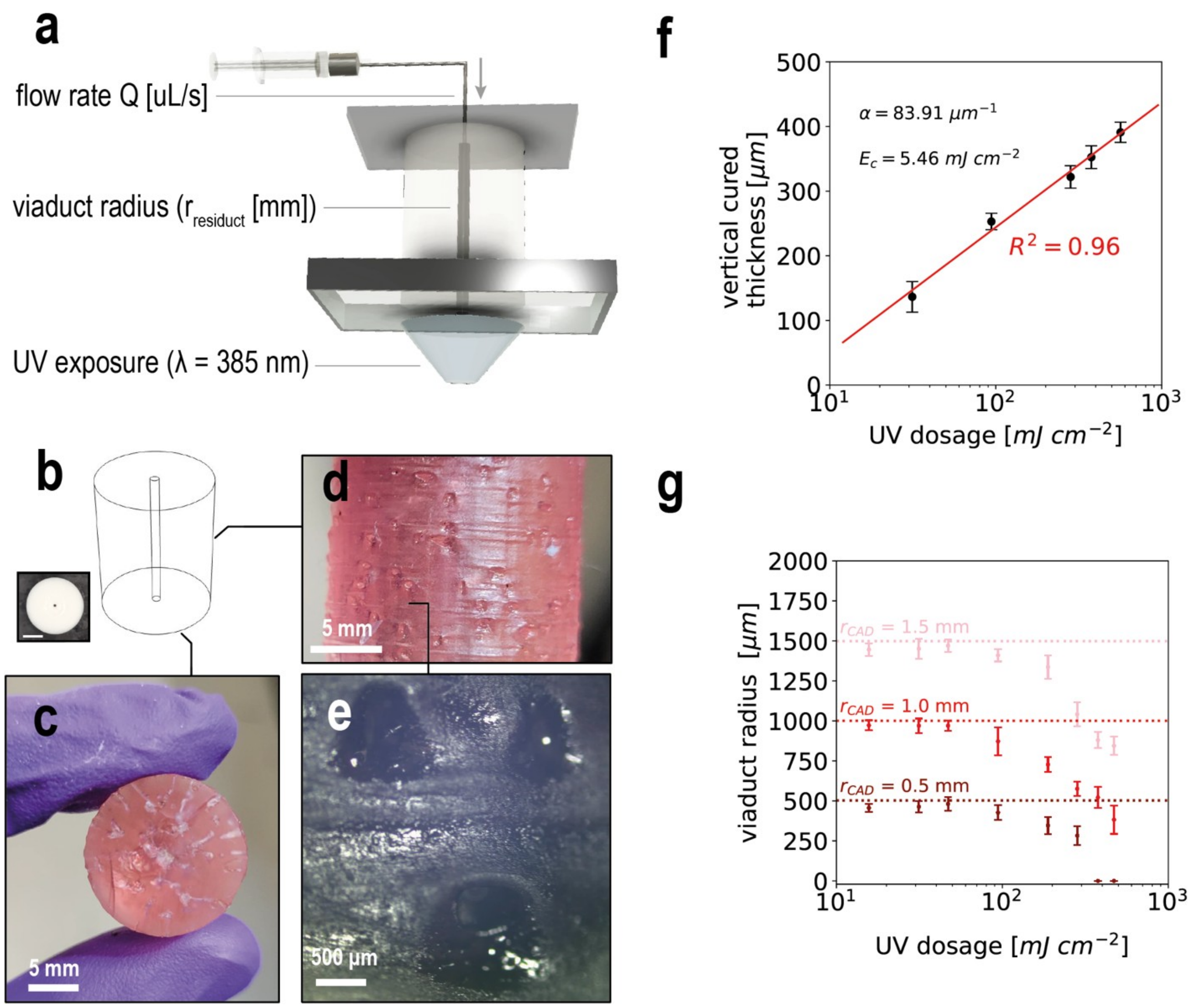

Figure S5: Viaduct design for iCLIP printing. (a) Test geometry for measuring viaduct radii, with UV projected through the oxygen permeable window into the vat while resin is injected through the part. **(b)** CAD geometry for evaluating viaduct radius, with a single channel internal to a solid cylinder. **(c-e)** Cross section (c), surface (d), and optical microscope image (e) of printed part showing stochastic and unprogrammed microfluidic channels resulting from resin flow through a laterally cured-through viaduct upon UV overexposure. **(f)** Quantified impact of UV dosage on vertical cured thickness, with measured α and E_c parameters from Beer's law indicated, in a range typical for CLIP printing. **(g)** Quantified impact of same UV light dosage range as in (c) on lateral cure through into the viaduct for viaducts with radii of 500 µm (**dark red**), 1000 µm (**red**), and 1500 µm (**pink**). Dotted lines indicate CAD specified viaduct radii. Error bars represent +/- one standard deviation from three independent experimental trials. Scale bars represent 5 mm, unless indicated otherwise.

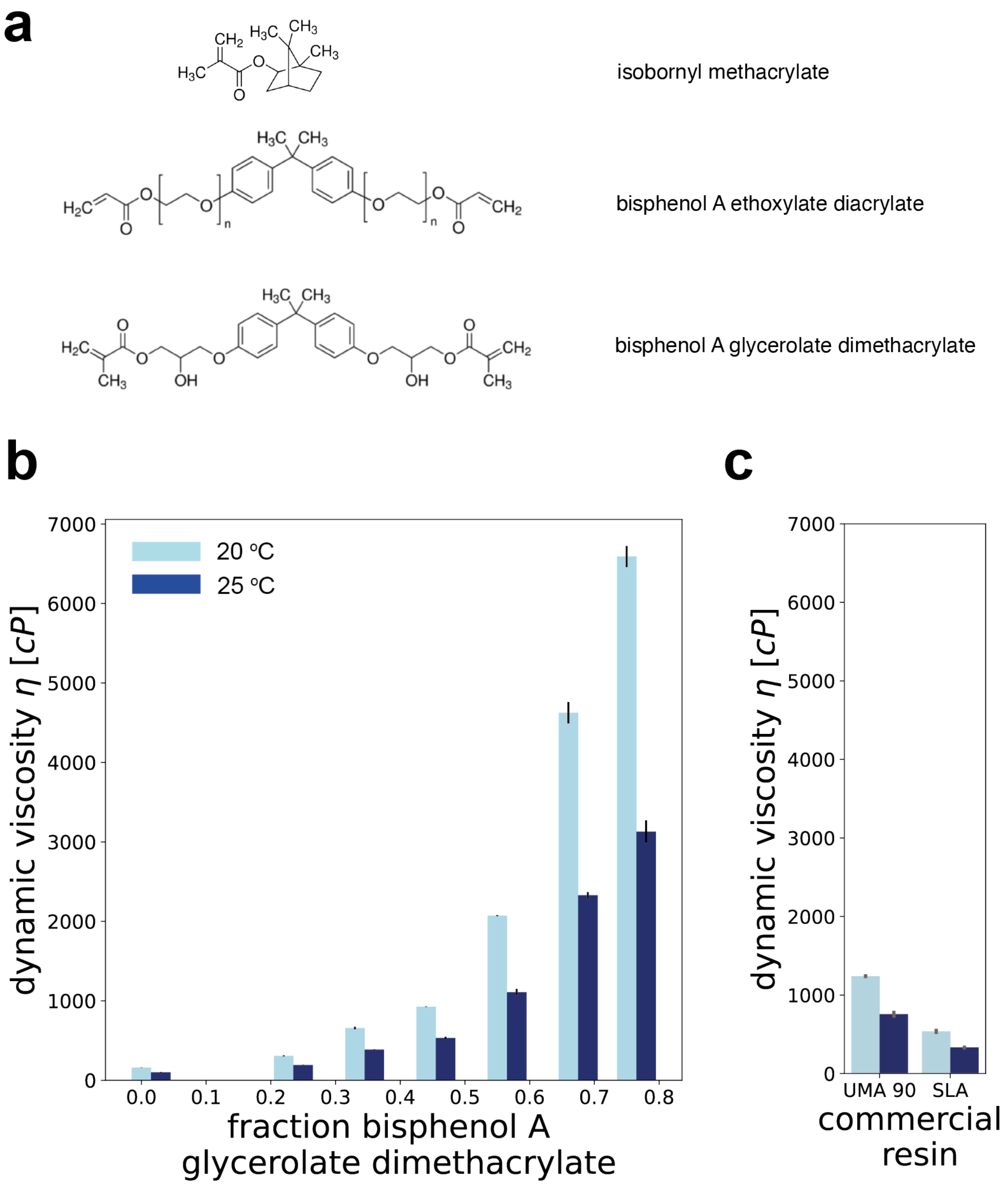

Figure S6: Rheological characterization of resins used to quantify printable viscosities in CLIP and iCLIP. (a) Components of the composite photocurable resin formulation, with a low viscosity diacrylate and high viscosity dimethacrylate. **(b)** Dependence of test resin viscosity on percentage bisphenol A glycerolate dimethacrylate. **Light blue** and **dark blue** indicate measured viscosities at 20 °C and 25 °C, respectively. **(c)** For comparison, viscosities of a standard CLIP resin from Carbon, Inc. (UMA 90) and a typical SLA resin from Formlabs, Inc.

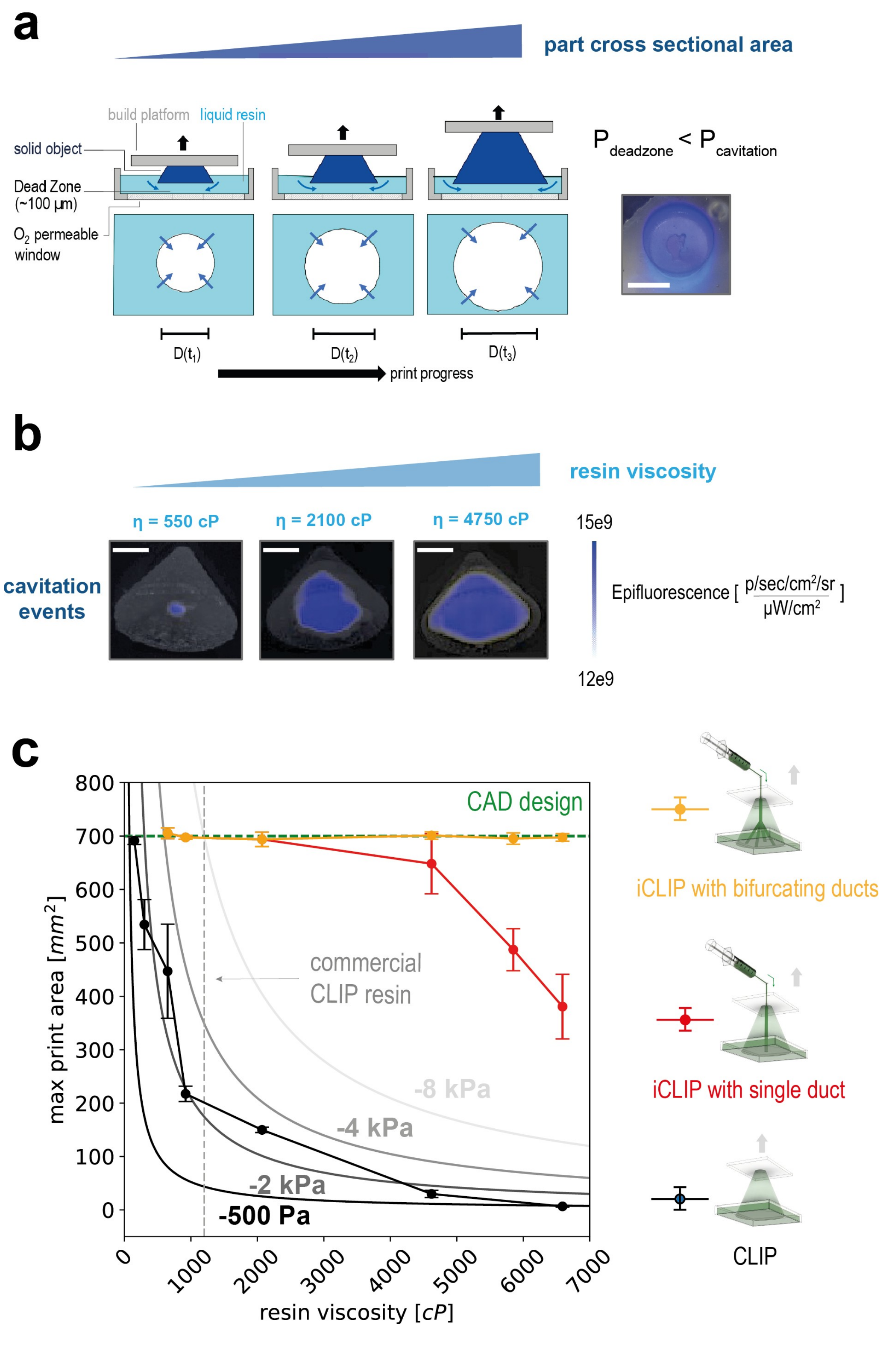

Figure S7: Printable viscosities by traditional CLIP and iCLIP. (a) Schematic illustrating mass transport limitations of traditional CLIP, with images of cavitation event viewed from underneath the vat during printing. **(b)** Images of cavitation events with resins of different viscosities after printing, with **blue** indicating hollowed out regions in an otherwise solid cone highlighted in **grey**. **(c)** Experimentally-quantified part radii at which cavitation failure occurs during CLIP and iCLIP with single or bifurcating viaducts, with comparison to theoretical minimum dead zone pressures during traditional CLIP for resins of varying viscosities printed over varying cross sectional areas. Lighter contours indicate higher (magnitude) negative pressures. Dotted green indicates target CAD area. Error bars denote +/- one standard deviation from three independent print trials.

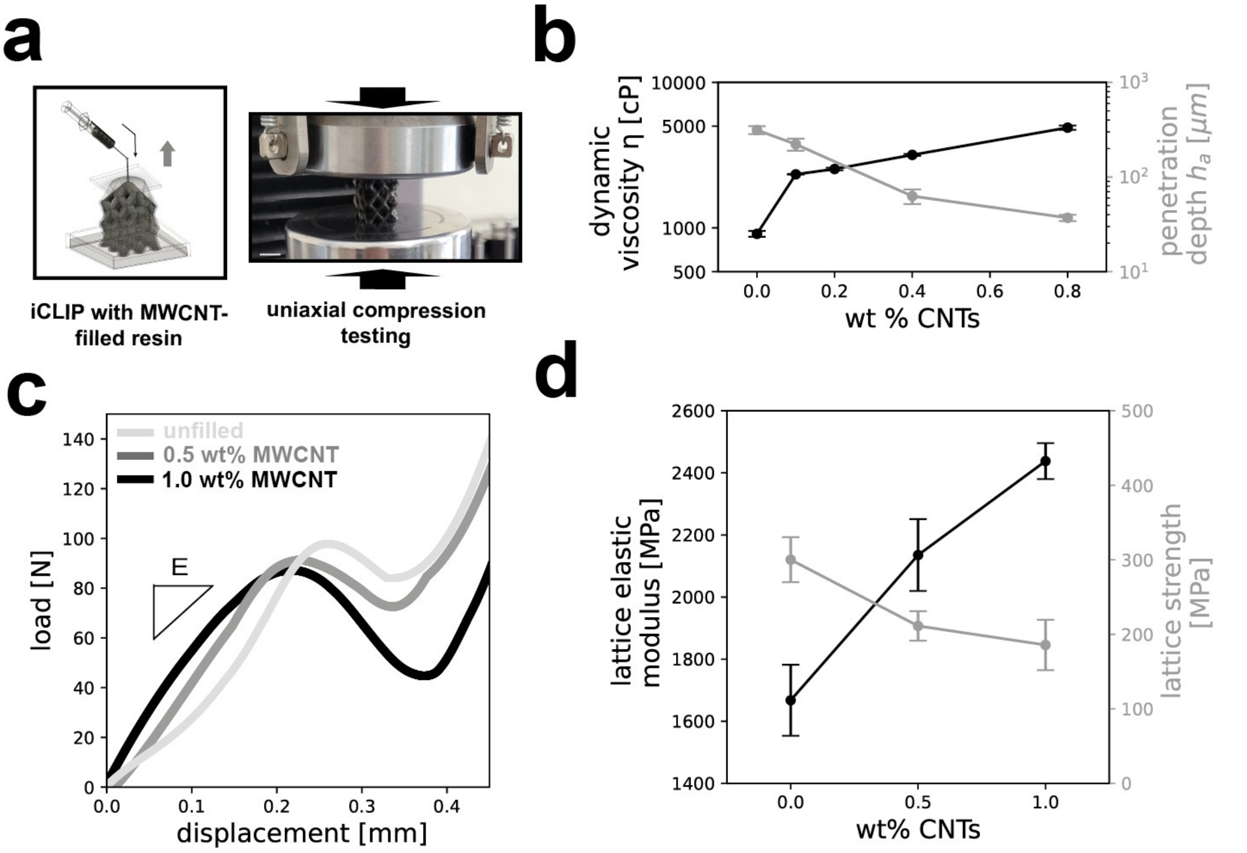

Figure S8: Printing with viscous composite resins by iCLIP. (a) iCLIP strategy for printing a lattice design with viscous MWCNT-filled resin. **(b)** Dynamic viscosity and UV penetration depth of resins with varying fiber volume fractions of CNTs, which are important factors to calibrate during printing. **(c)** Load-displacement curves under uniaxial compression for lattices with varying fiber volume fractions of CNTs. **(d)** Quantified elastic moduli and compressive strength of lattices with varying fiber volume fractions of CNTs under uniaxial compression. Scale bars denote 1 cm and error bars +/- one standard deviation from three experimental print trials.

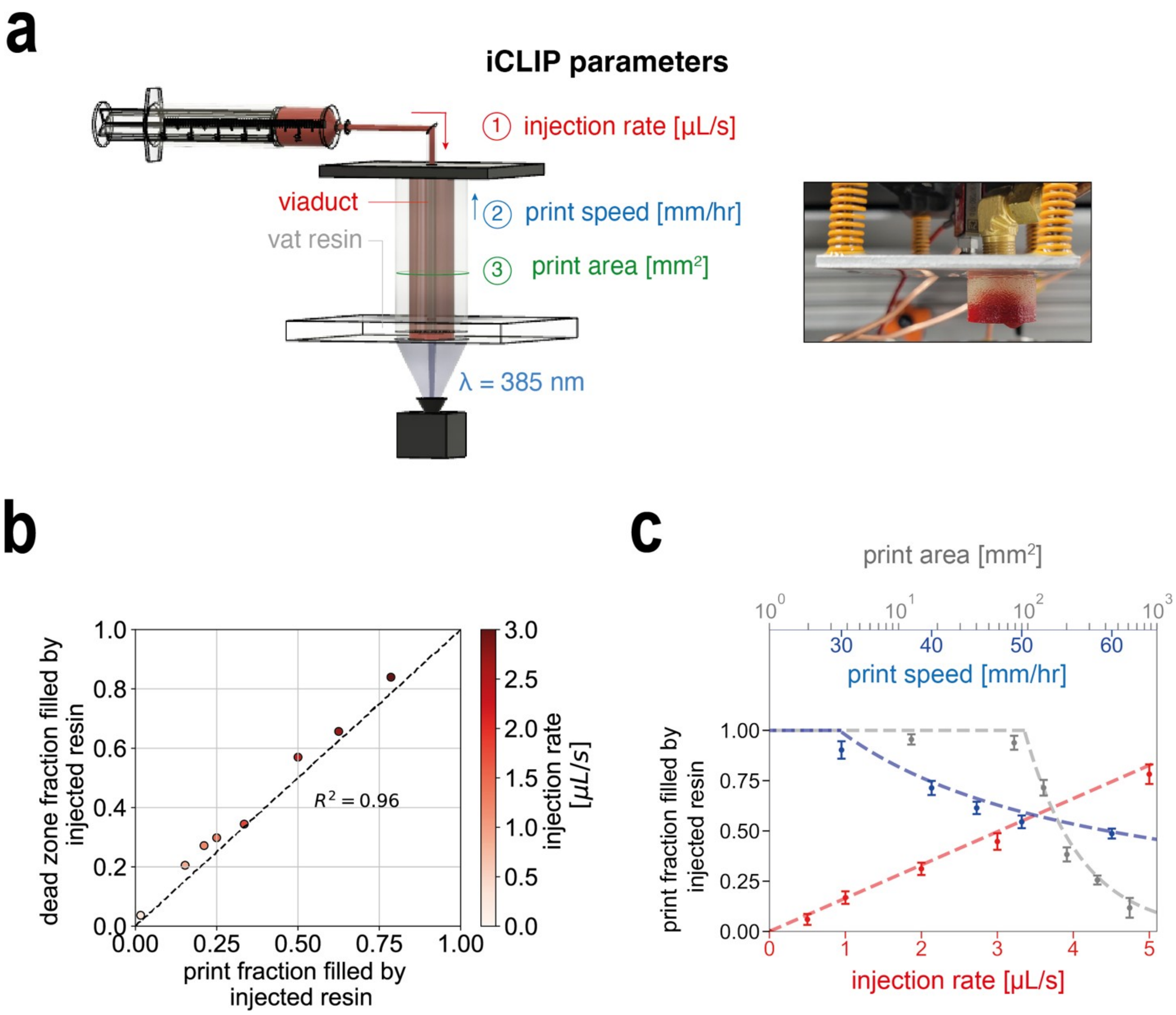

Figure S9: Calibration studies for multi-material iCLIP. (a) Test geometry for calibrating injection rates during iCLIP, with control parameters that can be tuned during an iCLIP print to adjust the fraction of vat-to-injected resin in a part. An example print result whereby injection rate is increased stepwise over the course of the print is shown to right. **(b)** Correlation between the fraction of dead zone filled by injected resin and fraction of print formed from injected resin. **(c)** Print parameter calibration experiments whereby one of three iCLIP print parameters is varied individually and the subsequent ratio of injected-to-vat resin in the final part measured. Error bars indicate +/- one standard deviation from three experimental print trials.

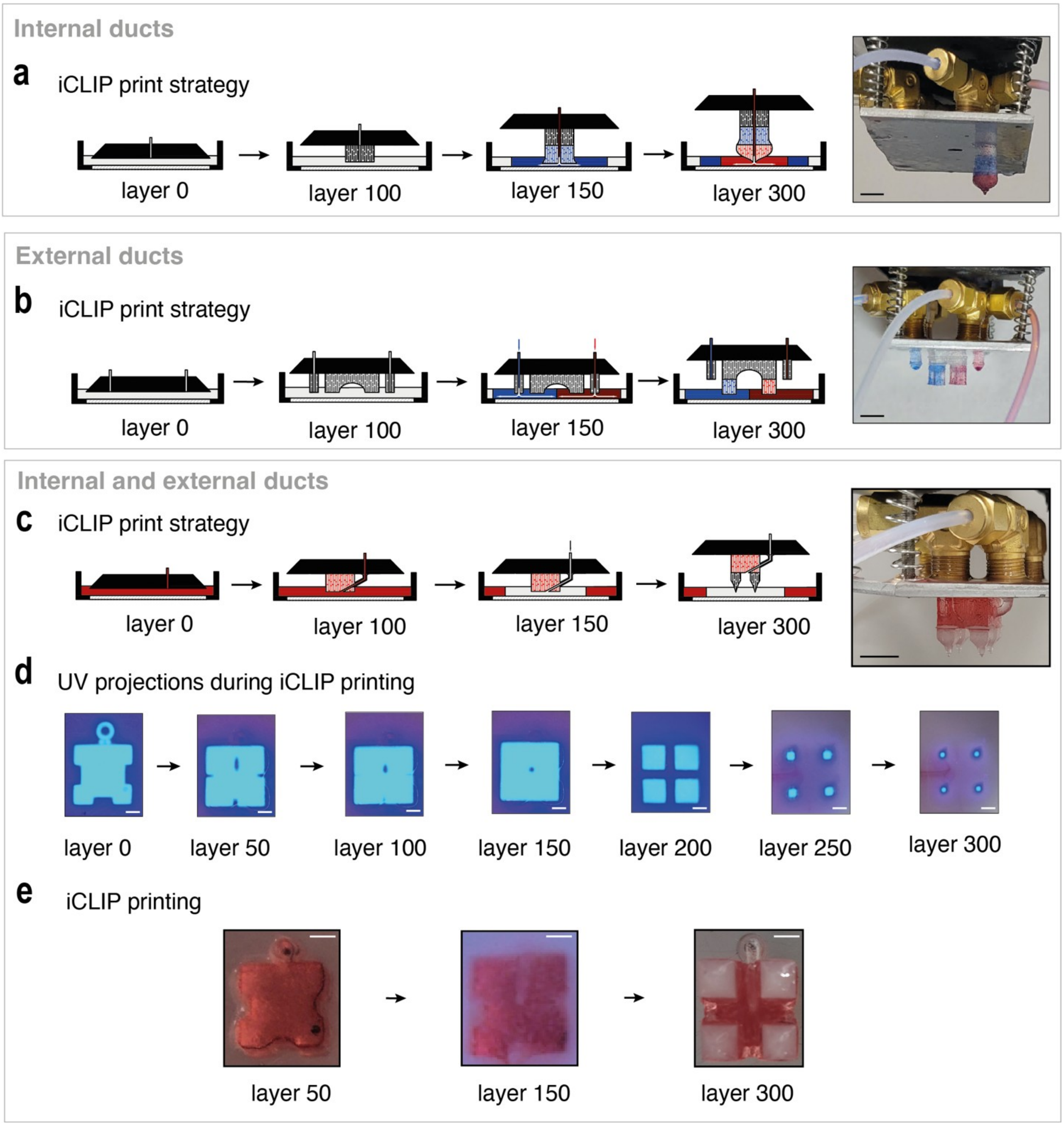

Figure S10: Design of internal and external duct geometries for multi-material iCLIP printing. (a) Illustrative example of a multimaterial iCLIP print script with a viaduct fully internal to the part, to print a model of St. Basil's Cathedral, with post-printing result shown to right. **(b)** Illustrative example of a multimaterial iCLIP print script with a viaduct fully external to the part, to print a model of the Arc de Triomphe, with post-printing result shown to right. **(c)** Illustrative example of a multimaterial iCLIP print script with a viaduct both internal and external to the part, to print a model of the Westminster Abbey, with post-printing result shown to right. An initially external viaduct merging with the part (c), reflected as a migrating circular region of no UV projections during printing **(d)**. The duct is visible as a conduit for white resin during and after printing **(e)**.

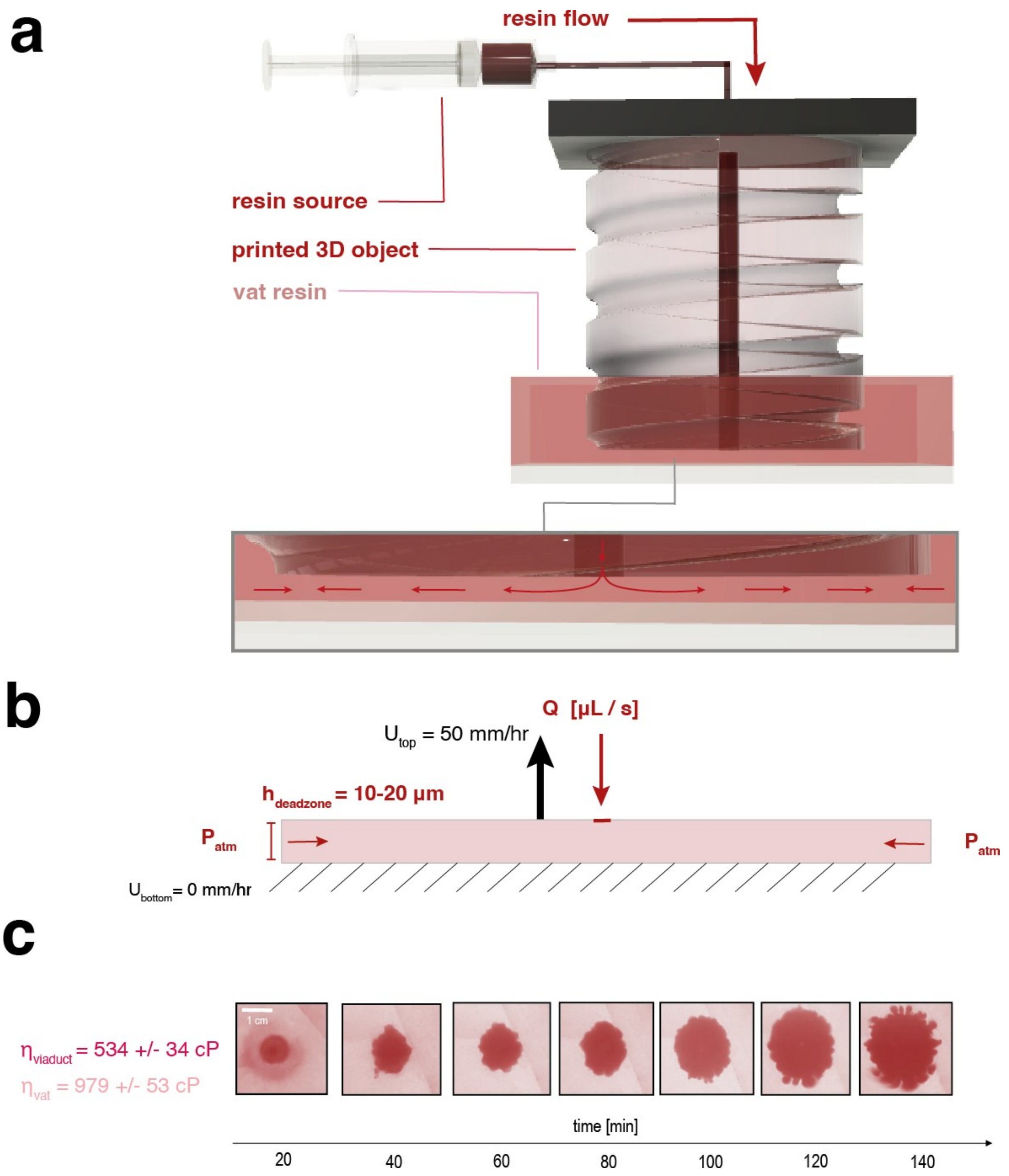

Figure S11: Viscous fingering during injection into a dead zone during multi-material iCLIP printing. (a) Schematic of iCLIP printing of a geometry with cylindrical cross sectional area. **(b)** Hele-Shaw cell model system for describing injection of a liquid into a thin gap with lifting upper surface. **(c)** During 2.5 hours of iCLIP printing of a cylindrical cross sectional geometry, instabilities in the flow boundary between a lower viscosity resin (**red**) injected through a central viaduct into the dead zone, and a higher viscosity resin (pink) present in the vat from the start of iCLIP printing, as viewed from beneath the optically transparent window by a digital imaging camera.

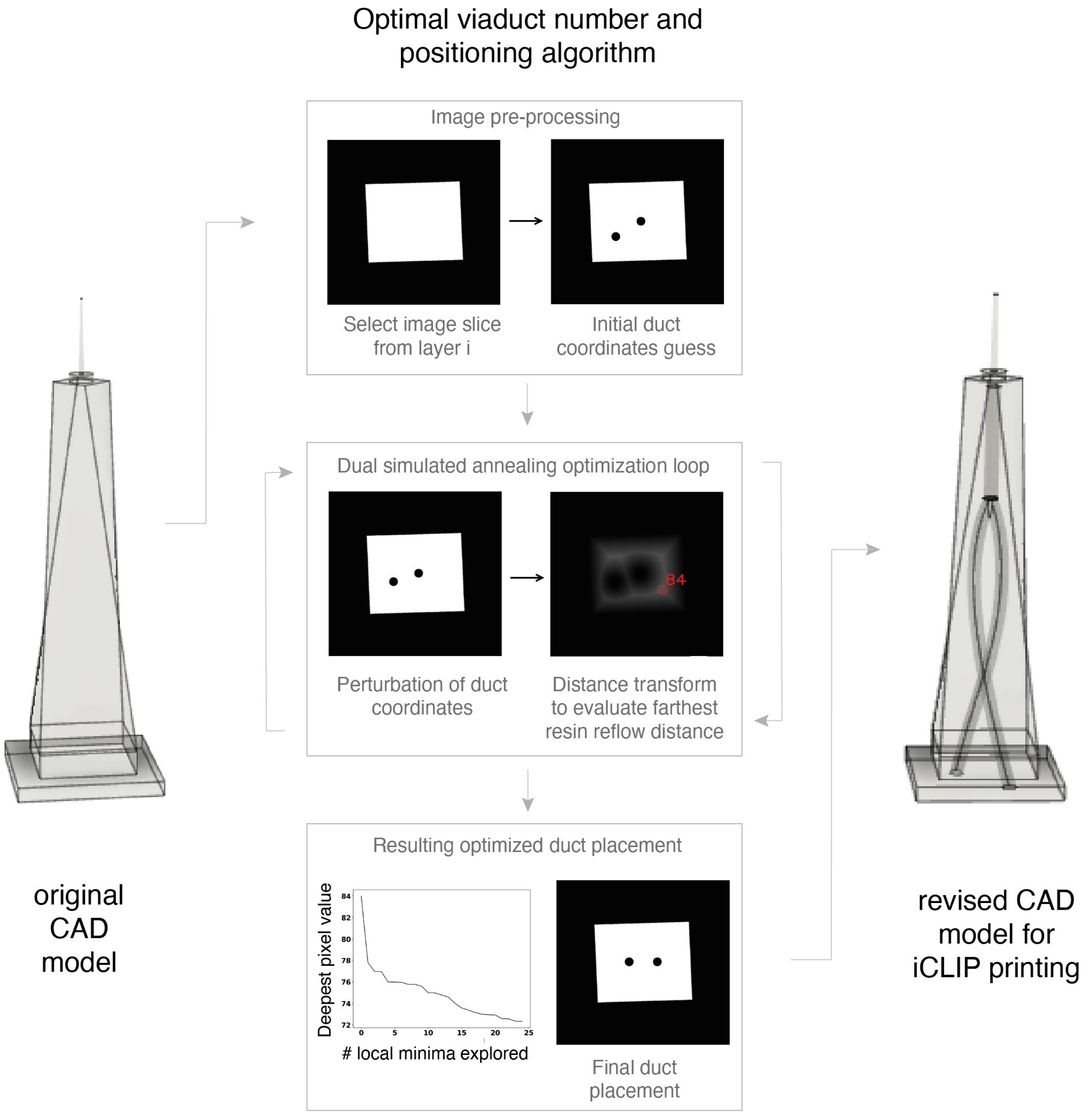

Figure S12: Dual simulated annealing algorithm for optimizing mass transport during iCLIP. Every digital z axis slice in an input CAD model is computationally analyzed with software that automatically incorporates viaducts to optimally distribute resin to alleviate suction forces and/or cavitation for high viscosity resins. The objective function to minimize by the dual simulated annealing algorithm implemented is the maximum resin reflow distance for a part cross sectional area. For a test geometry that both rotates and changes in area, a B spline smoothened arrangement of multiple, single, or no viaducts is produced.

REFERENCES AND NOTES

1. J. Huang, Q. Qin, J. Wang, A review of stereolithography: Processes and systems. *Processes* **8**, 1138 (2020)

2. J. Tumbleston, D. Shirvanyants, N. Ermoshkin, R. Janusziewicz, A. Johnson, D. Kelly, K. Chen, R. Pinschmidt, J. Rolland, A. Ermoshkin, E. Samulski, J. DeSimone, Continuous liquid interface production of 3D objects. *Science* **347**, 1349–1352 (2015).

3. R. Janusziewicz, J. Tumbleston, A. Quintanilla, S. Mecham, J. DeSimone, Layerless fabrication with continuous liquid interface production. *Proc. Natl. Acad. Sci. U.S.A.* **113**, 11703–11708 (2016).

4. A. Johnson, C. Caudill, J. Tumbleston, C. Bloomquist, K. Moga, A. Ermoshkin, D. Shirvanyants, S. Mecham, C. Luft, J. DeSimone, Single-step fabrication of computationally designed microneedles by continuous liquid interface production. *PLOS ONE* **11**, e0162518 (2016).

5. H. Ware, C. Sun, Method for attaining dimensionally accurate conditions for high-resolution three-dimensional printing ceramic composite structures using microclip process. *J. Micro Nano-Manuf.* **7**, 031001 (2019).

6. A. Bhanvadia, R. Farley, Y. Noh, T. Nishida, High-resolution stereolithography using a static liquid constrained interface. *Nat. Commun. Mater.* **2**, 41 (2021).

7. D. Walker, J. Hedrick, C. Mirkin, Rapid, large-volume, thermally controlled 3D printing using a mobile liquid interface. *Science* **80**, 360–364 (2019).

8. J. Bachmann, E. Gleis, G. Fruhmann, J. Riedelbauch, S. Schmölzer, O. Hinrichsen, Photo-differential scanning calorimetry parameter study of photopolymers used in digital light synthesis. *Addit. Manuf.* **3**, 41–53 (2022).

9. C. Caudill, J. Perry, S. Tian, J. Luft, J. DeSimone, Spatially controlled coating of continuous liquid interface production microneedles for transdermal protein delivery. *J. Control. Rel.* **284**, 122–132 (2018).

10. C. Caudill, J. Perry, I. Kimon, A. Tessema, B. Lee, B. Mecham, S. Tian, J. DeSimone, Transdermal vaccination via 3D-printed microneedles induces potent humoral and cellular immunity. *Proc. Natl. Acad. Sci. U.S.A.* **118**, e2102595118 (2021).

11. Y. Huang, C. Jiang, On-line force monitoring of platform ascending rapid prototyping system. *J. Mater. Process. Technol.* **159**, 257–264 (2005).

12. Q. Wang, Y. Sun, B. Guo, P. Li, Y. Li, CFD analysis and prediction of suction force during the pulling-up stage of the continuous liquid interface production process. *AIP Adv.* **9**, 015225 (2019).

13. Y. Pan, H. He, J. Xu, A. Feinerman, Study of separation force in constrained surface projection stereolithography. *Rapid Prototyp. J.* **23**, 353–361 (2017).

14. L. Wang, Y. Luo, Z. Yang, W. Dai, X. Liu, J. Yang, B. Lu, L. Chen, Accelerated refilling speed in rapid stereolithography based on nano-textured functional release film. *Addit. Manuf.* **29**, 100791 (2019).

15. X. Li, H. Mao, Y. Pan, Y. Chen, Mask video projection-based stereolithography with continuous resin flow. *J. Manuf. Sci. Eng. Trans. ASME* **141**, 081007 (2019).

16. J. Hardin, T. Ober, A. Valentine, J. Lewis, Microfluidic printheads for multimaterial 3D printing of viscoelastic inks. *Adv. Mater.* **27**, 3279–3284 (2015).

17. M. Skylar-Scott, J. Mueller, C. Visser, J. Lewis, Voxelated soft matter via multimaterial multinozzle 3D printing. *Nature* **575**, 330–335 (2019).

18. A. Rutz, K. Hyland, A. Jakus, W. Burghardt, R. Shah, A multimaterial bioink method for 3D printing tunable, cell-compatible hydrogels. *Adv. Mater.* **27**, 1607–1614 (2015).

19. D. Dendukuri, P. Panda, R. Haghgooie, J. Kim, T. Hatton, P. Doyle, Modeling of oxygen-inhibited free radical photopolymerization in a PDMS microfluidic device. *Macromolecules* **41**, 8547–8556 (2008).

20. E. Herbert, S. Balibar, F. Caupin, Cavitation pressure in water. *Phys. Rev. E Stat. Nonlin. Soft Matter Phys.* **74**, 041603 (2006).

21. J. Sandoval, R. Wicker, Functionalizing stereolithography resins: Effects of dispersed multi-walled carbon nanotubes on physical properties. *Rapid Prototyp. J.* **12**, 292–303 (2006).

22. G. Gonzalez, A. Chiappone, I. Roppolo, E. Fantino, V. Bertana, F. Perrucci, L. Scaltrito, F. Pirri, M. Sangermano, Development of 3D printable formulations containing CNT with enhanced electrical properties. *Polymer* **109**, 246–253 (2017).

23. T. Xiao, C. Qian, R. Yin, K. Wang, Y. Gao, F. Xuan, 3D printing of flexible strain sensor array based on UV-curable multiwalled carbon nanotube/elastomer composite. *Adv. Mater. Technol.* **6**, 2000745 (2021).

24. M. Porter, N. Ravikumar, F. Barthelat, F. R. Martini, 3D-printing and mechanics of bio-inspired articulated and multi-material structures. *J. Mech. Behav. Biomed. Mater.* **73**, 114–126 (2017).

25. G. Goh, H. Zhang, T. Chong, W. Yeong, 3D printing of multilayered and multimaterial electronics: A review. *Adv. Electron. Mater.* **7**, 2100445 (2021).

26. J. Mueller, K. Shea, Stepwise graded struts for maximizing energy absorption in lattices. *Extrem. Mech. Lett.* **25**, 7–15 (2018).

27. B. Khatri, M. Frey, A. Raouf-Fahmy, M. Scharla, T. Hanemann, Development of a multi-material stereolithography 3D printing device. *Micromachines* **11**, 532 (2020).

28. D. Han, C. Yang, N. Fang, H. Lee, Rapid multi-material 3D printing with projection micro-stereolithography using dynamic fluidic control. *Addit. Manuf.* **27**, 606–615 (2019).

29. J. Lind, T. Busbee, A. Valentine, F. Pasqualini, H. Yuan, M. Yadid, S. Park, A. Kotikian, A. Nesmith, P. Campbell, J. Vlassak, J. Lewis, K. Parker, Instrumented cardiac microphysiological devices via multimaterial three-dimensional printing. *Nat. Mater.* **16**, 303–308 (2017).

30. J. Boley, W. Rees, C. Lissandrello, L. Mahadevan, Shape-shifting structured lattices via multimaterial 4D printing. *Proc. Natl. Acad. Sci. U.S.A.* **116**, 20856–20862 (2019).

31. S. Uzel, R. Weeks, M. Eriksson, D. Kokkinis, J. Lewis, Multimaterial multinozzle adaptive 3D printing of soft materials. *Adv. Mater. Technol.* **1**, 2101710 (2022).

32. Q. Ge, A. H. Sakhaei, H. Lee, C. K. Dunn, N. X. Fang, M. L. Dunn, Multimaterial 4D printing with tailorable shape memory polymers. *Sci. Rep.* **6**, 31110 (2016).

33. J. Choi, H. Kim, R. Wicker, Multi-material stereolithography. *J. Mater. Process. Technol.* **211**, 318–328 (2011).

34. T. Islam, P. Gandhi, Viscous fingering in multiport hele shaw cell for controlled shaping of fluids. *Sci. Rep.* **7**, 1–9 (2017).

35. Submit buildings to iCLIParchitecture@outlook.com for our collection to demonstrate and challenge our multimaterial printing algorithms.

36. M. Shelley, F. Tian, K. Wlodarski, Hele–Shaw flow and pattern formation in a time-dependent gap. *Nonlinearity* **10**, 1471–1495 (1997).

37. L. Paterson, Radial fingering in a Hele Shaw cell. *J. Fluid Mech.* **113**, 513–529 (1981).

38. M. Mao, H. Jiankang, L. Xiaom B. Zhang, Q. Lei, Y. Liu, L. Dichen, The emerging frontiers and applications of high-resolution 3D printing. *Micromachines* **8**, 113 (2017).

39. S. Choo, S. Jin, J. Jung, Fabricating high-resolution and high-dimensional microneedle mold through the resolution improvement of stereolithography 3D printing. *Pharmaceutics* **14**, 766 (2022).

40. Z. Pritchard, M. Beer, R. Whelan, T. Scott, M. Burns, Modeling and correcting cure-through in continuous stereolithographic 3D printing. *Adv. Mater. Technol.* **4**, 1900700 (2019).

41. J. Bachmann, P. Obst, L. Knorr, S. Schmolzer, G. Fruhmann, G. Witt, T. Osswald, K. Wudy, O. Hinrichsen. Cavity vat photopolymerisation for additive manufacturing of polymer-composite 3D objects. *Commun. Mater.* **2**, 107 (2021).

42. D. Yadav, D. Chhabra, G. Kumar, A. Ahlawat, A. Phogat, Optimization of FDM 3D printing process parameters for multi-material using artificial neural network. *Mater. Today Proc.* **21**, 1583–1591 (2020).

43. G. Loke, R. Yuan, M. Rein, T. Khudiyev, Y. Jain, J. Joannopoulos, Y. Fink, Structured multimaterial filaments for 3D printing of optoelectronics. *Nat. Commun.* **101**, 1–10 (2019).

44. D. Kokkinis, M. Schaffner, A. Studart, Multimaterial magnetically assisted 3D printing of composite materials. *Nat. Commun.* **6**, 8643 (2015).

45. A. Georgopoulou, B. Vanderborght, F. Clemens, Multi-material 3D printing of thermoplastic elastomers for development of soft robotic structures with integrated sensor elements, in *Proceedings of the AMPA2020: Industrializing Additive Manufacturing*, M. Meboldt, C. Klahn, Eds. (Springer, 2021), pp. 67–81.

46. J. Yin, C. Lu, J. Fu, Y. Huang, Y. Zheng, Interfacial bonding during multi-material fused deposition modeling (FDM) process due to inter-molecular diffusion. *Mater. Des.* **150**, 104–112 (2018).

47. P. M. Mater, Prediction of in-plane stiffness of multi-material 3D printed laminate parts fabricated by FDM process using CLT and its mechanical behaviour under tensile load. *Mater. Today* **23**, 100955 (2020).

48. W. Wits, E. Amsterdam. Graded structures by multi-material mixing in laser powder bed fusion. *CIRP Ann.* **70**, 159–162 (2021).

49. M. Matsumoto, M. Shiomi, K. Osakada, F. Abe, Finite element analysis of single layer forming on metallic powder bed in rapid prototyping by selective laser processing. *Int. J. Mach. Tool Manuf.* **42**, 61–67 (2002).

50. Y. Chueh, X. Zhang, J. Chun-Ren Ke, Q. Li, C. Wei, L. Lin, Additive manufacturing of hybrid metal/polymer objects via multiple-material laser powder bed fusion. *Addit. Manuf.* **36**, 101465 (2020).

51. A. Renteria, V. Balcorta, C. Marquez, A. Rodriguez, I. Renteria-Marquez, J. Regis, B. Wilburn, S. Patterson, D. Espalin, T. Tseng, Y. Lin, Direct ink write multi-material printing of PDMS- BTO composites with MWCNT electrodes for flexible force sensors. *Flex. Print. Electron* **7**, 015001 (2022).

52. C. Xu, B. Quinn, L. Lebel, D. Therriault, G. L'Esperance, Multi-material direct ink writing (DIW) for complex 3D metallic structures with removable supports. *ACS Appl. Mater. Interfaces* **11**, 8499–8506 (2019).

53. D. Mccoul, S. Rosset, S. Schlatter, H. Shea, Inkjet 3D printing of UV and thermal cure silicone elastomers for dielectric elastomer actuators. *Smart Mater. Struct.* **26**, 125022 (2017).

54. R. Singh, R. Kumar, I. Farina, F. Colangelo, L. Feo, F. Fraternali, Multi-material additive manufacturing of sustainable innovative materials and structures. *Polymers* **11**, 62 (2019).

55. O. Yirmibesoglu, L. Simonsen, R. Manson, J. Davidson, K. Healy, Y. Menguc, T. Wallin, Multi-material direct ink writing of photocurable elastomeric foams. *Commun. Mater.* **21**, 1–14 (2021).

56. J. Vaithilingam, E. Saleh, R. Wildman, J. Hague, C. Tuck, Optimisation of substrate angles for multi-material and multi-functional inkjet printing. *Sci. Rep.* **8**, 9030 (2018).

57. T. Wallin, L. Simonsen, W. Pan, K. Wang, E. Giannelis, R. Shepherd, Y. Menguc. 3D printable tough silicone double networks. *Nat. Commun.* **111**, 1–10 (2020).

58. K. Kowsari, S. Akbari, D. Wang, N. Fang, Q. Ge, High-efficiency high-resolution multimaterial fabrication for digital light processing-based three-dimensional printing. *3D Print. Addit. Manuf.* **5**, 185–193 (2018).
